# Early Biomarker Signatures in Surgical Sepsis

**DOI:** 10.1016/j.jss.2022.04.052

**Published:** 2022-05-12

**Authors:** R.W.M.A. Madushani, Vishal Patel, Tyler Loftus, Yuanfang Ren, Han Jacob Li, Laura Velez, Quran Wu, Lasith Adhikari, Philip Efron, Mark Segal, Tezcan Ozrazgat-Baslanti, Parisa Rashidi, Azra Bihorac, Sepsis and Critical Illness Research Center Investigators

**Affiliations:** a University of Florida, Intelligent Critical Care Center, Gainesville, FL; b Department of Medicine, Division of Nephrology, Hypertension, and Renal Transplantation, University of Florida, Gainesville, Florida; c Department of Surgery, University of Florida, Gainesville, Florida; d Sepsis and Critical Illness Research Center, University of Florida, Gainesville, Florida; e J. Crayton Pruitt Family Department of Biomedical Engineering, University of Florida, Gainesville, Florida

**Keywords:** Biomarker, Clustering, Machine learning, Phenotyping, Sepsis, Unsupervised learning

## Abstract

**Introduction:**

Sepsis has complex, time-sensitive pathophysiology and important phenotypic subgroups. The objective of this study was to use machine learning analyses of blood and urine biomarker profiles to elucidate the pathophysiologic signatures of subgroups of surgical sepsis patients.

**Methods:**

This prospective cohort study included 243 surgical sepsis patients admitted to a quaternary care center between January 2015 and June 2017. We applied hierarchical clustering to clinical variables and 42 blood and urine biomarkers to identify phenotypic subgroups in a development cohort. Clinical characteristics and short-term and long-term outcomes were compared between clusters. A naїve Bayes classifier predicted cluster labels in a validation cohort.

**Results:**

The development cohort contained one cluster characterized by early organ dysfunction (cluster I, *n* = 18) and one cluster characterized by recovery (cluster II, *n* = 139). Cluster I was associated with higher Acute Physiologic Assessment and Chronic Health Evaluation II (30 *versus* 16, *P* < 0.001) and SOFA scores (13 *versus* 5, *P* < 0.001), greater prevalence of chronic cardiovascular and renal disease (*P* < 0.001) and septic shock (78% *versus* 17%, *P* < 0.001). Cluster I had higher mortality within 14 d of sepsis onset (11% *versus* 1.5%, *P* = 0.001) and within 1 y (44% *versus* 20%, *P* = 0.032), and higher incidence of chronic critical illness (61% *versus* 30%, *P* = 0.001). The Bayes classifier achieved 95% accuracy and identified two clusters that were similar to development cohort clusters.

**Conclusions:**

Machine learning analyses of clinical and biomarker variables identified an early organ dysfunction sepsis phenotype characterized by inflammation, renal dysfunction, endotheliopathy, and immunosuppression, as well as poor short-term and long-term clinical outcomes.

## Introduction

Sepsis, a dysregulated host response to infection leading to life-threatening organ dysfunction, is responsible for more than $20 billion in annual US healthcare expenditures and is associated with 18%–28% mortality.^1–3^ Optimal treatment involves early antibiotic administration, resuscitation, and source control of infection.^4,5^ With widespread adoption and implementation of this approach, sepsis mortality has decreased over time but remains unacceptably high. Sepsis is a broad syndrome defined and classified by clinical criteria not necessarily reflective of underlying pathological processes, and most sepsis drugtrials have failed. Given the heterogeneity of sepsis, identifying subgroups of sepsis patients with unique pathophysiological signatures and treatment responses may be necessary to develop successful targeted therapies.^6^

Grouping routinely collected clinical variables and biomarkers from sepsis patients with clustering, an unsupervised machine learning technique, will allow the identification of entirely novel phenotypes.^6–8^ Unsupervised machine learning has been used previously to identify subgroups in other complex syndromes, such as acute respiratory distress syndrome (ARDS).^9^ Given the complex pathophysiology of sepsis and heterogeneity among sepsis patients, a battery of physiologic measurements of organ dysfunction obtained during routine clinical care and blood and urine metabolic and immunologic biomarkers are available to allow early identification of patients at risk for poor short-term and long-term outcomes.^6^ Illness severity scoring systems such as the Sequential Organ Failure Assessment (SOFA) and Acute Physiologic Assessment and Chronic Health Evaluation II (APACHE II) scores and inflammatory and immunosuppressive biomarkers can forecast mortality within 24 h of sepsis onset and thus may help predict the underlying pathophysiology of various sepsis phenotypes.^7,10–14^ Validating the utility and accuracy of an unsupervised learning model on routinely available clinical variables may provide a basis for future studies differentiating sepsis management efficacy based on phenotype.

We used machine learning analyses of clinical variables, as well as blood and urine biomarker profiles to identify phenotypic subgroups in a prospective, longitudinal cohort of surgical sepsis patients. Our objective was to elucidate the pathophysiologic signatures of phenotypic subgroups, with the rationale that a deeper understanding of sepsis phenotypes can inform the development of targeted therapies.

## Methods

### Patient recruitment

**Fig. 1 –:**
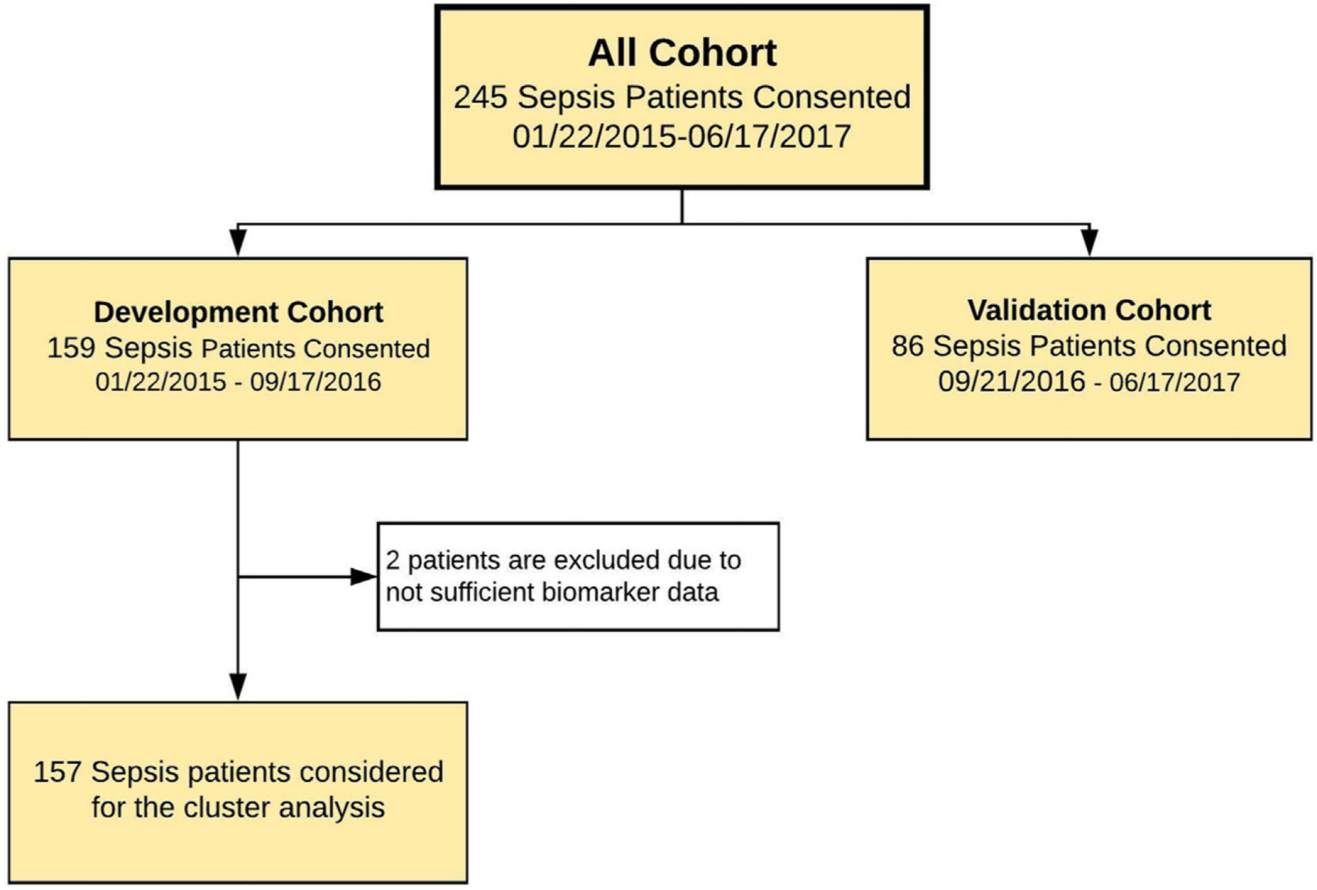
Patient enrollment and inclusion flowchart.

Sepsis patients were prospectively recruited between January 2015 and June 2017 from the Persistent Inflammation and Immunosuppression in Sepsis (PICS; NCT02276066) prospective longitudinal study cohort of surgical patients with sepsis. Study protocols were finalized,^15^ and ethics approval was obtained from the University of Florida Institutional Review Board (IRB201400611) prior to patient enrollment. All study participants or their surrogate decision-maker provided written informed consent. Inclusion criteria for the sepsis cohort were admission to the surgical intensive care unit (ICU), age 18 y or greater, and a clinical diagnosis of sepsis by attending intensivist in surgical ICU with subsequent initiation of the computerized sepsis protocol.^16^ Patients with pre-existing immunosuppression and those with advanced liver or heart disease were excluded to avoid their potentially confounding effects on the natural history of sepsis and the subsequent development of chronic critical illness.^15^ The final sepsis diagnosis was clinically adjudicated by investigators during weekly adjudication meetings according to consensus criteria.^17^ Among 243 patients meeting enrollment criteria with sufficient biomarker data, 157 were included in the development cohort and 86 were included in the validation cohort. Patients were allocated to development or validation cohorts, based on split in time, as illustrated in Figure 1. This study was registered at Clinicaltrials.gov (NCT02276417).

### Generation of biomarker signatures

**Fig. 2 –:**
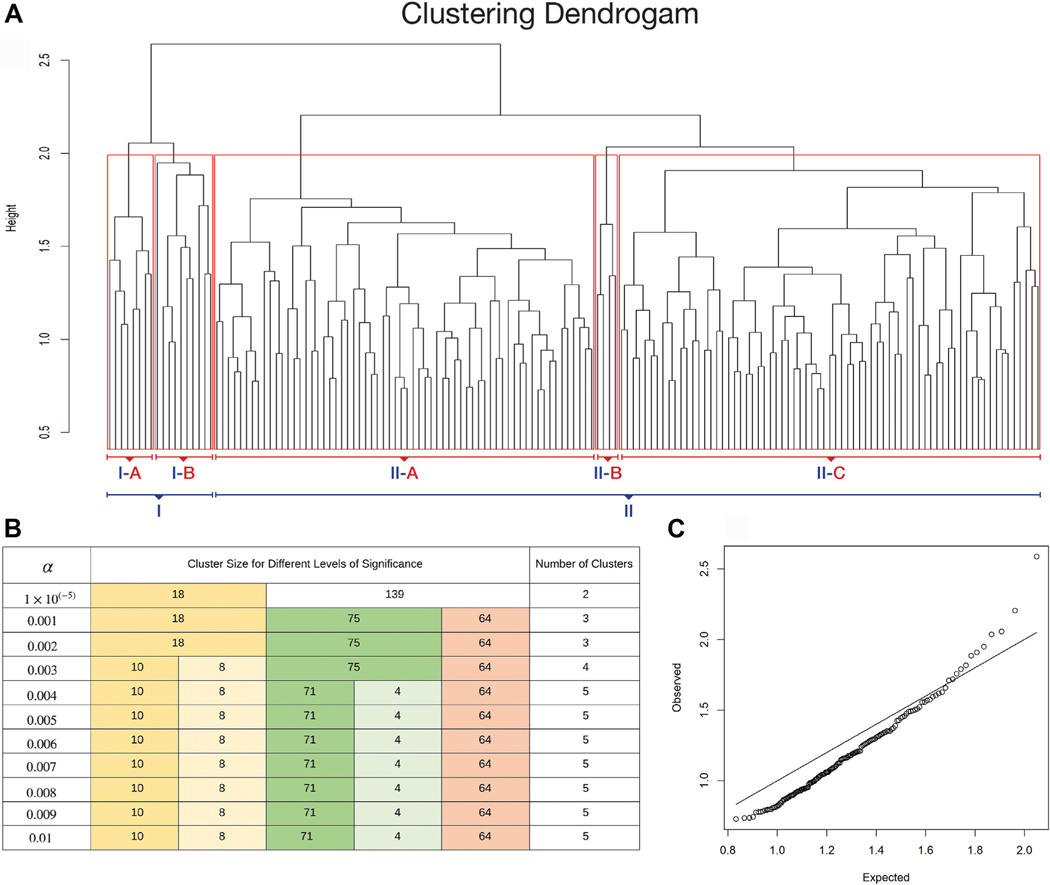
Clustering dendrogram with significance. (A) Dendrogram showing the subject profile arrangement from the hierarchical clustering using the complete linkage method with normalized distances by the number of features 42. (B) Cluster composition for different levels of significance α. Colors track clusters that are robust with increasing significance levels. (C) QQ-plot displaying the observed and expected distances used in hierarchical clustering (height of branch nodes). Departure from the diagonal line suggests that there are significant clusters in the data.

The clustering algorithms used 58 laboratory and vital sign measurements obtained within 24 h of sepsis onset, as listed in Supplement Table E1. Missing values were imputed using the median value of each biomarker for the entire study population. Standardized values of 42 biomarkers, as well as laboratory values, vital signs, subject age, and Charlson comorbidity index, were used to cluster 157 patients into groups with similar clinical and biomarker profiles using an agglomerative hierarchical clustering with complete linkage based on Euclidean distance, that is, the distance between two points in high-dimensional space.^18–20^ Agglomerative nesting is a type of hierarchical clustering in which the algorithm starts by treating each object as a singleton cluster, and then pairs of clusters are successively merged until all clusters have been merged into one cluster containing all objects. A dendrogram obtained from hierarchical clustering using the vector of distances denoted by *D*_0_ is shown in Figure 2A.

### Cluster number and identification

A resampling approach was used to identify clusters by cutting the dendrogram as previously described.^5,21^ In this resampling method, we generate a reference distribution *D*_*ref*_ for *D*_0_ under the null hypothesis that there would be no significant clusters in the data by randomly mixing the biomarker values of patients for each biomarker and then performing hierarchical clustering on the permuted dataset. We repeat the resampling procedure ten times, and the reference distribution *D*_*ref*_ is calculated by taking the average of the distances used for hierarchical clustering in each set. The plot of the observed (*D*_0_) and expected distances (*D*_*ref*_) is displayed in Figure 2C. Significant clusters were detected by cutting the dendrogram at the 99.6 percentile with α = 0.4% of the reference distribution, identifying five clusters, which are unlikely to appear in random data. Visual representations, as well as clinical characteristics and outcomes of five clusters, were observed to determine the number of clusters for primary analyses. After investigating the clinical characteristics and outcomes, due to observing only minor differences between two clusters (71 and 64) and in order to avoid even smaller sample sizes spread, we decided to report two main clusters, which corresponds to cutting the dendrogram at the significance level α = 0.001%, in the primary analysis. The sensitivity of cluster robustness to different significance levels is illustrated in Figure 2B.

The dependency of clusters on all variables was tested using ‘leave one feature out’ replication (Supplement Table E2),^21^ in which the cluster analysis was repeated by removing one of the 44 biomarkers at a time, and then hierarchical cluster analysis and selection was performed using the same method as in the primary analysis of two clusters with the significance level of 0.004. Concordance between primary analysis clusters was assessed by Spearman correlations of the cluster labels, summarized in Supplement Table E2, with low correlation indicating a change in cluster assignments after exclusion of the feature, suggesting the importance of the feature (Supplementary Methods).

A naїve Bayes classifier was trained on the development cohort and used to assign patients to derived clusters in the validation cohort. A naїve Bayes classifier is a model based upon the Bayes theorem obtained by using a set of discriminant functions and estimating relevant probabilities from a training set. In this case, the classifier uses the set of pre-existing cluster prevalence and the presence of each biomarker signature to predict the cluster membership of new patients.

For each patient, we created a biomarker mosaic using the gene expression dynamics inspector (GEDI) that creates the biomarker mosaics using a self-organizing map algorithm.^22,23^ We compared clinical characteristics and outcomes between the two main clusters using Fisher’s exact test for categorical variables and Student’s *t*-test or Wilcoxon rank-sum test for continuous variables as appropriate.

### Definition of outcomes

All outcomes were compared among clusters and between cohorts. The primary outcomes were in-hospital and 1-year mortality. Chronic critical illness (CCI) was defined as an ICU length of stay of 14 d or more with evidence of persistent organ dysfunction, determined using components of the SOFA score (cardiovascular SOFA ≥1, or score in any other organ system ≥2). Non-CCI patients were those who did not meet the criteria for CCI or early death (death within 14 d of sepsis onset).^24^ Other outcomes included hospital-free, ICU-free, and mechanical ventilation-free days within 28 d of sepsis onset. Exact dates and times were used to calculate the hospital length of stay, ICU length of stay, and duration of mechanical ventilation. Hospital-free, ICU-free, mechanical ventilation-free, and organ dysfunction free-days within 28 d of sepsis onset were calculated by subtracting the number of days for each outcome from the lesser of 28 d or the number of days between sepsis onset and death. The Social Security Death Index database was used to confirm death dates and obtain death dates for patients who were lost to follow-up.

## Results

### Cluster identification

Cluster analysis identified two main clusters (Cluster I, Cluster II) and five statistically distinct clusters (I (A), I (B), II (A), II (B), II (C)) (*P* < 0.005, Fig. 2A and B). We focus on the two largest clusters: cluster I and cluster II (A, B, and C), which were obtained by cutting the dendrogram at significance level α = 0.00001. A QQ plot of expected and observed normalized distances among clusters shows similar clusters producing distances smaller than expected by chance and dissimilar clusters producing distances larger than expected by chance (Fig. 2C).

### Clinical characteristics of development cohort clusters

Table 1 summarizes the clinical characteristics of 18 patients in cluster I and 139 patients in cluster II. Cluster I, characterized by early organ dysfunction, contained a greater proportion of septic shock patients (78% *versus* 17%, *P* < 0.001) and higher median APACHE II scores (30 *versus* 16, *P* < 0.001) and SOFA scores (13 *versus* 5, *P* < 0.001). Higher APACHE II scores in cluster I was primarily attributable to higher acute physiology scores (23 *versus* 12, *P* < 0.001) rather than the age or chronic health scores. Two chronic diseases differentiated between clusters: cardiovascular and renal diseases (*P* < 0.001). Forty-seven percent of patients in cluster I had chronic kidney disease, and 39% had congestive heart failure. In cluster II, 11% patients had chronic kidney disease, and 14% had congestive heart failure. There were no significant differences in age, gender, race, body mass index, or smoking history between clusters.

### Outcome characteristics of development cohort clusters

Associations between cluster biomarker signatures and clinical outcomes are shown in Table 2. Cluster I had higher mortality within 14 d of sepsis onset (11% *versus* 2%, *P* = 0.001), during admission (33% *versus* 5%, *P* = 0.001), and within 1 y (44% *versus* 20%, *P* = 0.032). Cluster I had a longer median ICU length of stay (15 *versus* 6 d, *P* < 0.001) and fewer ICU-free days (3 *versus* 22 d, *P* < 0.001). The incidence of acute kidney injury (AKI) was nearly two-fold higher in cluster I (78% *versus* 48%, *P* < 0.001). Thirty-nine percent of the cluster I patients required renal replacement therapy during admission, while only 5% of cluster II patients required renal replacement therapy (*P* < 0.001). Overall, 72% of the cluster I patients suffered early death or developed chronic critical illness, compared with 32% of cluster II patients.

### Biomarker characteristics of development cohort clusters

**Fig. 3 –:**
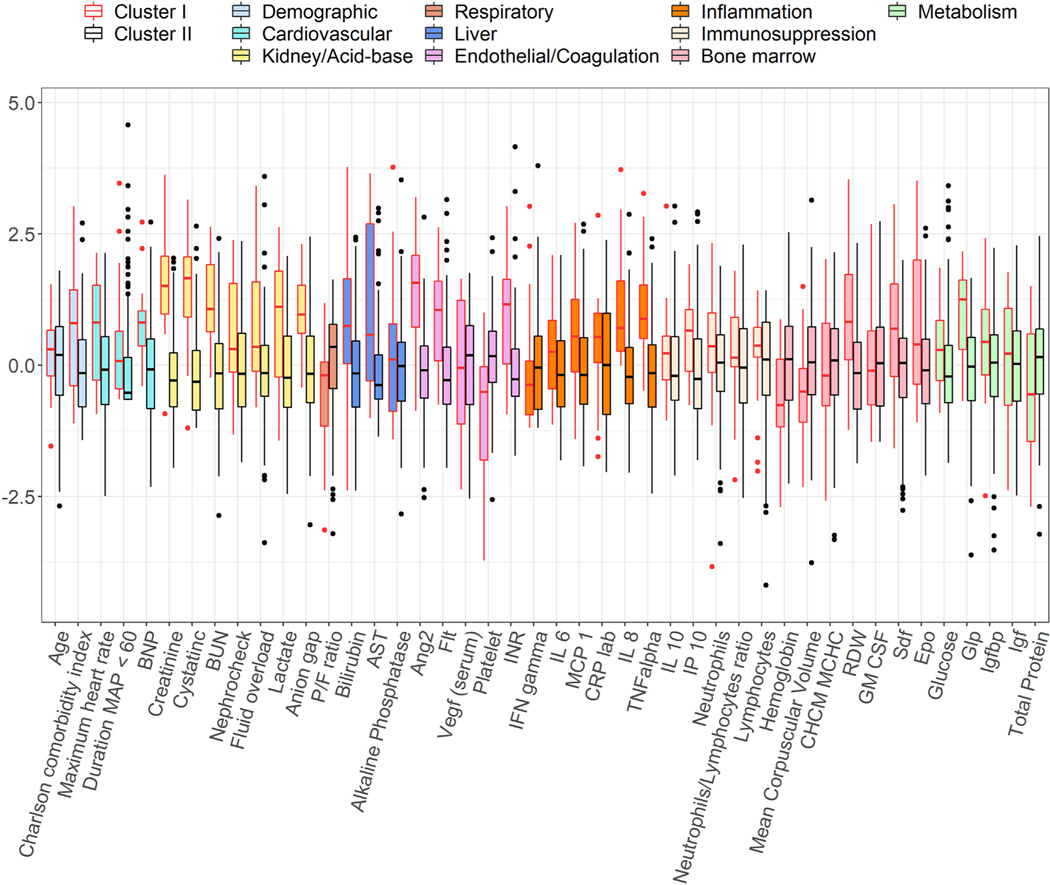
Distributions of biomarkers by clusters. Side-by-side boxplots show the distributions of the standardized biomarker values across clusters. Each color represents a group of biomarkers based on their functionality. In each plot, the horizontal dash line represents the average value of the standardized biomarker in the whole cohort.

**Fig. 4 –:**
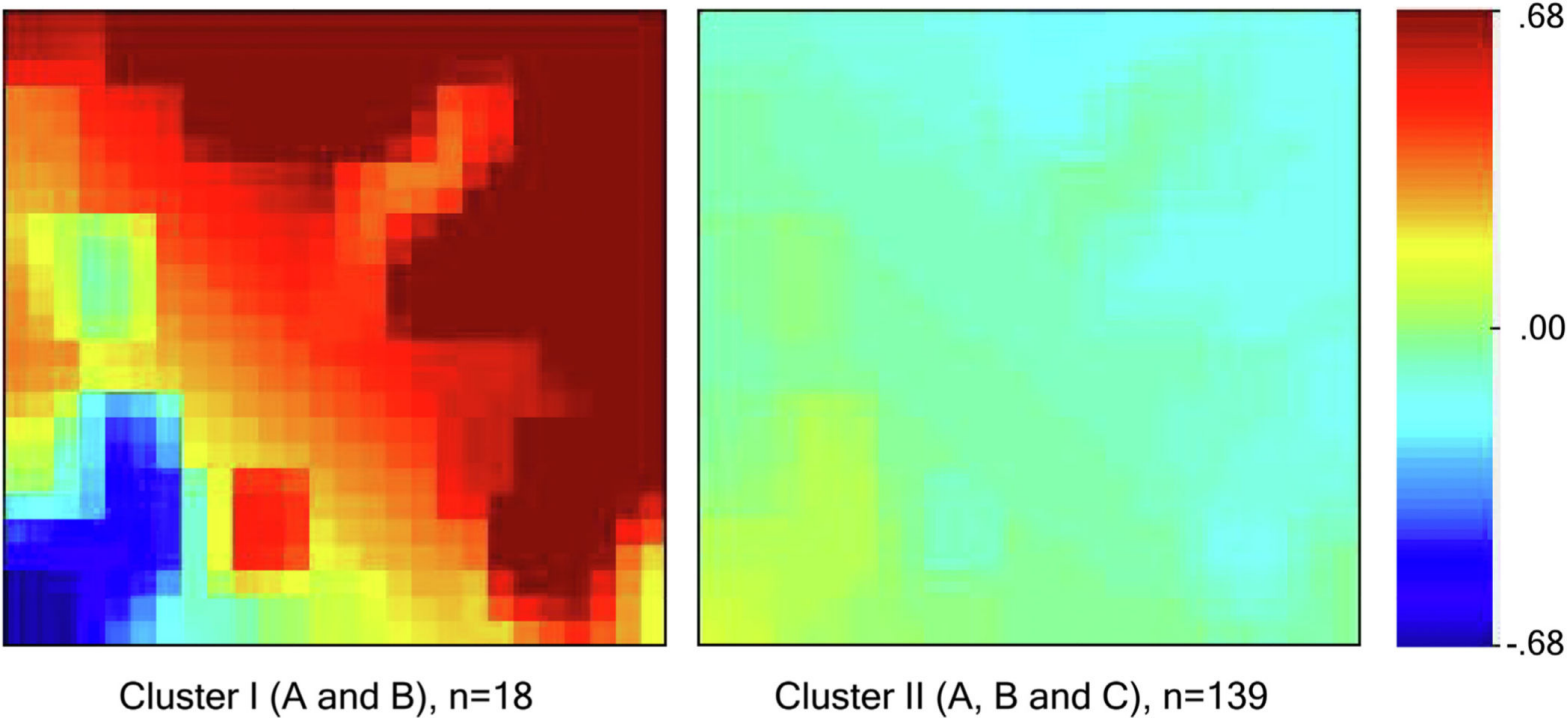
Average biomarker mosaics for patients using a self-organizing map for each of the two main clusters. Average biomarker mosaics of a specific cluster illustrate the average value of biomarker values for patients within that cluster. Red color correlates with increased biomarker expression, and blue color correlates with decreased biomarker expression compared to mean values for the development cohort, which is illustrated by the green color.

Visual cluster representation using principal component analysis shows that clusters are separated in biomarker space (Supplement Fig E1). Biomarker distributions for the two main clusters are illustrated in Figure 3 and Supplement Figure E2, respectively. Similar has been shown for all five clusters in Supplement Figure E3. The reference mosaics representing all 44 biomarker variables for the two main clusters and for all five clusters are illustrated in Figure 4 and Supplement Figure E4, respectively. Supplement Figure E4 illustrates mosaics for two example patients within each of the five clusters. Cluster I was characterized by high heterogeneity among biomarker expression (i.e., some biomarkers are drastically increased or decreased compared to the average values of the development cohort).

Table 3 summarizes differences in biomarker values between clusters I and II. Cluster I exhibited a biomarker profile consistent with inflammation, immunosuppression, and metabolic dysregulation.

Cluster I demonstrated early renal dysfunction. Cluster I had higher median serum creatinine (2.8 *versus* 0.96 mg/dL, *P* < 0.001), cystatin C (2.3 *versus* 0.8 mg/dL *P* < 0.001), and blood urea nitrogen (44 *versus* 19 mg/dL, *P* < 0.001). Endotheliopathy may have contributed, as angiopoietin 2 levels were 3–4 times higher in cluster I (27 *versus* 8 ng/mL *P* < 0.001), and fms-like tyrosine kinase levels were 2–3 times higher in cluster I (442 *versus* 174 pg/mL, *P* < 0.001). Other renal and acid-base parameters such as anion gap, lactate, Nephrocheck scores, and fluid overload volumes were significantly worse in cluster I (Fig. 3, Table 3). Consistent with renal dysfunction and volume overload, median brain natriuretic peptide was nearly three times higher in cluster I (3922 *versus* 1024 pg/mL, *P* < 0.001).

Other biomarkers in cluster I that were significantly elevated included bilirubin, aspartate aminotransferase, international normalized ratio, interleukin 8, tumor necrosis factor-alpha, monocyte chemoattractant protein-1, and glucagon-like peptide, suggesting an inflammatory state with hepatic dysfunction. In addition to the biomarkers used in the cluster analysis, soluble programmed death-ligand 1 was included to represent immunosuppression and was significantly higher in cluster I, as was interferon gamma-induced protein 10, suggesting immunosuppression.

In the leave-one-out analysis to assess the relative importance of each biomarker in assigning cluster labels (Supplement Table E2), absolute cluster concordance correlation coefficients ranged from 0.03 when platelet counts were left out to 0.66 when maximum heart rate was left out.

### Predicting cluster labels in the validation cohort

To determine whether clusters with similar characteristics could be identified in an independent validation cohort, we trained a naїve Bayes classifier on the development cohort to predict cluster labels in the validation cohort of 86 patients. Some biomarker values (e.g., glucagon-like peptide, NephroCheck) were not available for patients in the validation cohort. As the derivation model was highly sensitive to the absence of singular features from leave-one-out analysis, a naїve Bayes classification model, which is more robust to missing variables, was used to minimize the impact of missing data. The classifier achieved 95% accuracy for leave-one-feature out cross-validation in the development cohort. The classifier was then applied to the validation cohort. The classifier assigned 29 validation cohort patients to cluster I and 57 patients to cluster II. Compared with development clusters, the same patterns of clinical characteristics, outcomes, and biomarker values were observed (see Supplement Tables E3, E4, and E5). Cluster I had higher APACHE II scores (25 *versus* 17, *P* < 0.001) and total Acute Physiology Scores (20 *versus* 14, *P* < 0.001) compared with cluster II. Cluster I also had higher SOFA scores at sepsis onset (9 *versus* 5, *P* < 0.001) with higher cardiovascular (3 *versus* 1, *P* < 0.001) and renal (3 *versus* 0, *P* < 0.001) scores at sepsis onset. These were associated with poor short-term and long-term outcomes. Cluster I (*n* = 29) had higher hospital mortality (*P* = 0.03) and 1-year mortality (*P* < 0.001) and had more than twice as many ICU days (*P* = 0.03). The incidence of early death or chronic critical illness was significantly higher among cluster I patients (75% *versus* 27%, *P* < 0.001).

Characteristics of the development and validation cohorts were compared in order to determine if clusters identified in the development cohort were identifiable in an independent validation cohort due to the cohorts being similar (Supplement Tables E6, E7, and E8). This analysis revealed several important differences between cohort characteristics. The validation cohort contained a smaller proportion of patients with congestive heart failure (5% *versus* 17%, *P* < 0.01), different primary sources for sepsis (*P* = 0.02), and higher maximum renal SOFA scores within 24 h of sepsis onset (1 *versus* 0, *P* = 0.02). The validation cohort had higher in-hospital mortality (18% *versus* 8%, *P* = 0.03) and a greater burden of kidney disease and magnitude of AKI. Consistent with these clinical outcomes, the validation cohort had a biomarker profile consistent with kidney disease, including higher serum creatinine and cystatin C. It also featured differences in endothelial dysfunction evident by higher angiopoietin 2 and fms-like tyrosine kinase, a proinflammatory immunosuppressed state manifested as higher tumor necrosis factor-alpha, interferon-gamma, soluble programmed death-ligand 1. Therefore, the identification of early dysfunctional and recovery clusters is reproducible in cohorts that may have different clinical characteristics and biomarker profiles compared to the development cohort.

## Discussion

Using 42 blood and urine biomarkers and routinely collected clinical data, we identified two major clusters of patients with surgical sepsis. Inflammatory, renal, and endothelial biomarkers that differentiated cluster I from cluster II included interleukin 8, tumor necrosis factor-alpha, serum creatinine, cystatin C, blood urea nitrogen, anion gap, fluid overload, lactate, angiopoietin 2, and fms-like tyrosine kinase. These biomarkers contributed significantly to differences in composite biomarker mosaics in both clusters, suggesting that systemic inflammation, renal dysfunction, and endotheliopathy were primary drivers of cluster differentiation. Leave-one-out analysis suggested that all biomarkers contributed significantly to primary cluster assignment because excluding any of these biomarkers would result in different cluster assignments. Although agglomerative hierarchical clustering is dependent on data dimensionality (i.e., number of clustering biomarkers), making it more likely that leave-one-out analysis produces different results as a statistical artifact, we found that a naïve Bayes classifier, which is more robust to dimensional changes, was able to successfully reproduce similar clusters on a validation cohort, suggesting that biomarker profiles reflect underlying septic pathology.

Consistent with these biomarker profiles, we observed a greater prevalence of chronic renal and cardiovascular disease in cluster I. Furthermore, cluster I had severe early multiorgan failure with a disproportionately high incidence of cardiovascular and renal disease. These results are consistent with a previous study by Garcia-Obregon *et al.* (2018),^25^ in which a similar panel of ten proteins in a prospective cohort of 85 patients predicted sepsis with cardiovascular dysfunction. In addition, cluster I patients had an immunosuppressive phenotype manifest as increased interferon gamma-inducible protein 10 and soluble programmed death-ligand 1. Prior work has not consistently demonstrated concomitant inflammation and immunosuppression, as was observed in our study. In an analysis of peripheral blood leukocyte gene expression among patients with sepsis due to pneumonia, Davenport *et al.* (2016)^26^ found that inflammation and immunosuppression occur in separate, distinct sepsis response signatures. However, a meta-analysis of 949 sepsis patients using hierarchical clustering demonstrated significant inflammation and immunosuppression in early sepsis, similar to results from our study.^27^

The unique biologic signatures of clusters I and II corresponded to different illness severity and clinical outcomes. The acute physiology component of the APACHE score differentiated between clusters, similar to results obtained by Knox *et al.* (2015)^7^ Cluster I had higher Charlson comorbidity indices, suggesting that they had a greater chronic disease burden prior to the onset of sepsis, but the difference in the acute physiological score between clusters I and II was of greater magnitude. Cluster I had a higher incidence of septic shock, which could be explained by the cardiovascular physiological derangement. Cluster I had worse clinical outcomes with higher early and 1-year mortality rates. The 5% in-hospital mortality and 20% 1-year mortality rates observed in Cluster II are lower than the mortality rates observed in other studies of contemporary populations with sepsis; this may be attributable to the early preserved hemostasis biomarker profile and phenotype observed in Cluster II.

High heterogeneity in clinical and biomarker characteristics across phenotypes among sepsis patients may provide insight regarding failed sepsis drug trials. Seymour *et al.* (2019)^6^ demonstrated that there are subgroups of sepsis patients with unique responses to treatments, offering compelling evidence that broadly applied monotherapies for sepsis and septic shock are likely to continue to fail. Our study does not address the hypothesis that different sepsis phenotypes have different treatment responses but supports the hypothesis that clustering analysis can identify hidden patterns and structures within sepsis patient data, identifying phenotypes with distinct short-term and long-term outcomes. Also, clustering analysis may help improve the performance of prediction and risk-stratification for these outcomes by building separate prediction models for each cluster. These observations were made in a prospective study of a relatively small group of patients, suggesting that it is feasible to perform these clustering techniques in clinical settings.

We demonstrate that the application of machine learning analytic methods to a battery of routine clinical, physiologic measurements of organ dysfunction in concert with blood and urine biomarkers of renal function, tissue perfusion, inflammation, and immunosuppression can identify surgical sepsis phenotypes. Although these are done in a small cohort as a proof-of-concept, these findings demonstrate the potential benefit of machine learning-derived clusters of sepsis in treatment. For example, as we utilize routine biomarkers, all procured within hours of ICU admission, machine learning may rapidly identify septic patients at high risk of AKI requiring aggressive resuscitation or renal replacement therapy. Furthermore, as Cluster I is defined by an inflammatory phenotype, these patients may benefit from anti-inflammatory therapy, which can be administered quickly and potentially improve surgical outcomes. Similar approaches have demonstrated efficacy for phenotyping other critical illnesses, suggesting broader implications for understanding the host response to critical illness.^28^ Calfee *et al.* (2018)^9^ performed a secondary, latent class analysis to identify acute respiratory distress syndrome subphenotypes in a multicenter, randomized controlled trial database. This analysis identified distinct hyperinflammatory and hypoinflammatory phenotypes with different biological features and clinical outcomes. Perhaps more importantly, the administration of simvastatin conferred a survival advantage that was specific to the hyperinflammatory group, suggesting that the identification of phenotypes can guide patient-specific treatments. Similarly, Antcliffe *et al.* (2019)^29^ performed a secondary analysis of a randomized clinical trial database of sepsis patients to determine whether phenotypes of sepsis patients have unique responses to corticosteroid administration. Patients with an immunocompetent phenotype had increased mortality after corticosteroid administration compared with placebo. These findings suggest that phenotyping techniques not only elucidate underlying pathophysiology but are also associated with unique treatment responses. Machine learning techniques may be ideal for representing complex disease syndromes like sepsis and acute respiratory distress syndrome because their underlying pathophysiology is beyond the reach of additive and linear statistical approaches.^30^

### Study limitations

Our study has several limitations. First, we used a small data sample of surgical patients from a single institution, limiting the power and generalizability of these findings. In addition, perhaps the greatest value of phenotyping is the ability to assess responses to targeted therapies. Accomplishing this objective would require the application of biomarker signatures to data from randomized controlled trials, which is feasible but beyond the scope of this study. Second, the method we used to evaluate the importance of each biomarker to the clusters (leave-one-out analysis) was susceptible to false positives. The merge step of agglomerative clustering cannot be reversed and is dependent on the distance, and subsequently the dimensionality of the data. By reducing the dimensions of the dataset in leave-one-out analysis, reperforming agglomerative clustering is more likely to find different results. Despite the reliance of leave-one-out on statistical methods, we still report these findings (1) to show the magnitude of dependence for each variable and (2) to account for the possibility that some biomarkers may be unimportant even given the clustering method’s limitations. Likewise, we used a naїve Bayes classifier to predict clusters in our validation cohort, which makes an assumption of data independence. Although some collected variables, such as BUN and Cr, do not satisfy the independence assumption, naїve Bayes was chosen for its simplicity and outperformance of other alternatives even in some cases where the independence assumption is not met.^31,32^ Third, we use a single time point, rather than longitudinal data. Although we focus on capturing patient profiles shortly after sepsis diagnosis and exclude patients with advanced liver or heart disease, our approach did not account for the evolution of sepsis prior to and after ICU admission. Thus, it is possible that our clusters represent differences in the evolution of sepsis, with Cluster II representing a resolved state. However, given that our study focuses on exploring the ability of machine learning to derive sepsis phenotypes, longitudinal analyses were deemed out of scope. Finally, we did not perform an external validation of our derived clusters using a large dataset, and this should be considered in the interpretation of our findings.

## Conclusions

Machine learning analyses of clinical and biomarker variables identified an early organ dysfunction sepsis phenotype characterized by inflammation, renal dysfunction, endotheliopathy, and immunosuppression, as well as poor short-term and long-term clinical outcomes. These efforts to elucidate the pathophysiologic signatures of phenotypic subgroups may provide a deeper understanding of sepsis phenotypes that can inform the development of targeted therapies.

## Supplementary Material

1

2

3

4

5

6

7

8

9

10

figsE4

figsE3

figsE2

figsE1

## Figures and Tables

**Table 1 –:** Clinical characteristics of patients in two main clusters.

Clinical characteristics	Cluster I early disrupted homeostasis (*N* = 18)	Cluster II early preserved homeostasis (*N* = 139)	*P* value

Demographics			
Age, median (25th, 75th)	64 (56, 69)	62 (50, 70)	0.35
Male gender, *n* (%)	11 (61)	74 (53)	0.62
Race, *n* (%)			
White	15 (83)	124 (89)	0.07
African American	1 (6)	12 (9)	
American Indian	0 (0)	1 (0.7)	
Transferred from another hospital, *n* (%)	8 (44)	64 (46)	1.00
Weight (kg), median (25th, 75th)	98 (64, 136)	86 (71, 103)	0.28
Body Mass index, median (25th, 75th)	35 (21, 45)	29 (25, 34)	0.36
Comorbidities, n (%)			
Chronic kidney disease*	7 (47)	15 (11)	**0.001**
Congestive heart failure	7 (39)	20 (14)	**0.02**
Chronic obstructive pulmonary disease^†^	2 (12)	17 (12)	1.00
Diabetes mellitus^†^	8 (47)	45 (33)	0.28
Hypertension	13 (76)	81 (59)	0.20
Smoking history, *n* (%)			
Current	4 (24)	57 (42)	0.21
Former	1 (6)	15 (11)	
Never	12 (71)	64 (47)	
Characteristics of sepsis episode			
Sepsis severity, *n* (%)			
Sepsis	0 (0)	57 (41)	**< 0.001**
Severe sepsis	4 (22)	58 (42)	
Septic shock	14 (78)	24 (17)	
Acuity scores in the first 24 h of sepsis onset, median (25th, 75th)			
APACHE II	30 (26, 39)	16 (11, 21)	**< 0.001**
Total acute physiology score	23 (20, 34)	12 (8, 17)	**< 0.001**
Age points	4 (3, 5)	3 (2, 5)	0.19
Chronic health points	0 (0, 2)	0 (0, 0)	**0.001**
SOFA	13 (11, 15)	5 (2, 8)	**< 0.001**
Respiratory system	3 (2, 3)	0 (0, 3)	**< 0.001**
Central nervous system	3 (2, 4)	1 (0, 2)	**0.002**
Cardiovascular system	3 (3, 4)	1 (0, 1)	**< 0.001**
Liver	1 (0, 2)	0 (0, 0)	**< 0.001**
Coagulation	0.5 (0, 2)	0 (0, 0)	**0.002**
Renal	3 (2, 4)	0 (0, 1)	**< 0.001**

Abbreviations: APACHE II = Acute Physiology and Chronic Health Evaluation II score; SOFA = Sequential Organ Failure Assessment. Pairs that are significant with *P* values at 0.05 level are boldfaced.

* Percentages calculated after removing end-stage renal disease (ESRD) patients from the cohort.

† Due to missing values, the percentages were calculated based on the available data.

**Table 2 –:** Clinical outcomes among cluster I and II development cohort patients.

Clinical outcomes	Cluster I early disrupted homeostasis (*N* = 18)	Cluster II early preserved homeostasis (*N* = 131)*	*P* value

Hospital mortality, *n* (%)	6 (33)	6 (5)	**0.001**
One year mortality, *n* (%)^†,‡^	8/18 (44)	24/122 (20)	**0.032**
One year mortality among survivors, *n* (%)^‡^	2/12 (17)	18/116 (16)	>0.999
Chronic critical illness (CCI), *n* (%)			**0.001**
Early death	2 (11)	2 (2)	
CCI	11 (61)	39 (30)	
Non-CCI	5 (28)	90 (69)	
Kidney disease, *n* (%)	17 (94)	67 (52)	**<0.001**
ESKD	3 (17)	0 (0)	
CKD, no AKI	0 (0)	5 (4)	
AKI and CKD	7 (39)	10 (8)	
AKI, no CKD	7 (39)	52 (40)	
No renal disease	1 (6)	64 (50)	
Acute kidney injury severity, *n* (%)	14 (78)	62 (47)	**<0.001**
Stage 1	2 (11)	33 (25)	
Stage 2	5 (28)	18 (14)	
Stage 3	7 (39)	11 (8)	
Renal replacement therapy (RRT), n (%)	7 (39)	6 (5)	**<0.001**
Duration of RRT (days)^§^, median (25th, 75th)	13 (2, 50)	13 (8, 27)	0.67
RRT-free days to day 28, median (25th, 75th)	10 (0, 28)	28 (28, 28)	**<0.001**
Renal recovery at discharge, n (%)^║^	2 (14)	44 (71)	**<0.001**
Hospital days, median (25th, 75th)	20 (14, 36)	17 (8, 28)	0.16
Hospital-free days to day 28, median (25th, 75th)	0 (0, 8)	10 (0, 19)	**0.006**
Days in intensive care unit, median (25th, 75th)	15 (8, 24)	6 (3, 15)	**0.001**
ICU-free days to day 28, median (25th, 75th)	3 (0, 15)	22 (13, 25)	**<0.001**
Need for mechanical ventilation, *n* (%)	18 (100)	87 (66)	**0.002**
Days on mechanical ventilator^¶^, median (25th, 75th)	7 (5, 16)	5 (2, 10)	**0.04**
MV-free days to day 28, median (25th, 75th)	16 (0, 23)	26 (21, 28)	**<0.001**
SOFA organ dysfunction-free days to day 28	0 (0, 18)	19 (10, 24)	**<0.001**
Discharged home, *n* (%)	2 (11)	71 (54)	**0.001**
Readmission or death within 30 d of initial discharge, *n* (%)	4 (22)	32 (24)	>0.999

Abbreviations: ESKD = end-stage kidney disease; CKD = chronic kidney disease; CCI = chronic critical illness; ICU = intensive care unit; MV = mechanical ventilation; RRT = renal replacement therapy; SOFA = Sequential Organ Failure Assessment. Pairs that are significant with *P* values at 0.05 level are boldfaced.

* Hospital outcome data were not available for some patients due to the withdrawal of patients from the study before hospital discharge.

† Due to missing values percentages were calculated based on available.

‡ Twelve-month data were not available for some patients due to the withdrawal of patients from the study before the 12-month follow-up.

§ among patients who required RRT.

║ among patients who had AKI.

¶ among patients who required mechanical ventilation.

**Table 3 –:** Characteristics of biomarkers measured within 24 h of sepsis onset that differentiated between development cohort clusters.

Biomarkers within 24 h of sepsis onset	Cluster I early disrupted homeostasis (*N* = 18)	Cluster II early preserved homeostasis (*N* = 139)	*P* value

Cardiovascular			
Brain natriuretic peptide (BNP), pg/mL	3922 (1943,5559)	1024 (322,2501)	**<0.001**
Duration mean arterial pressure (MAP) < 60, mmHg (min)^†^	109 (27,202)	18 (0,120)	**0.01**
Maximum heart rate (beats per min) within first 24 h from the sepsis onset	134 (115,147)	119 (108,130)	**0.02**
Kidney			
Serum creatinine, mg/dL	2.8 (2, 3.9)	0.96 (0.7, 1.3)	**<0.001**
Cystatin C, mg/dL	2.3 (1.5, 2.9)	0.8 (0.6, 1.1)	**<0.001**
Blood urea nitrogen (BUN), mg/dL	44 (31, 84)	19 (12, 28)	**<0.001**
Anion gap, mmol/L	21 (19, 24)	16 (14, 19)	**<0.001**
Fluid overload, %	11 (7, 21)	6 (3, 11)	**0.002**
Lactate, mmol/L	4 (1.7, 6)	1.8 (1.3, 2.9)	**0.002**
Nephrocheck	0.7 (0.38, 3.44)	0.36 (0.15, 1.01)	**0.008**
Respiratory			
Ratio of partial pressure arterial oxygen and fraction of inspired oxygen (PaO2/FiO2), mmHg	249 (134,293)	349 (210,457)	**0.01**
Liver			
Bilirubin, mg/dL	1.6 (0.8, 3.5)	0.7 (0.4, 1.2)	**<0.001**
Aspartate aminotransferase (AST) test (SGOT), U/L	68 (26,666)	25 (19, 46)	**0.001**
Endothelial function and coagulation			
Angiopoietin-2 (Ang2), ng/mL	27 (13, 39)	8 (5, 11)	**<0.001**
Fms-related tyrosine (Flt), pg/mL	442 (215,644)	174 (127,267)	**<0.001**
International normalized ratio (INR)	1.95 (1.5, 2.2)	1.4 (1.3, 1.6)	**<0.001**
Platelet count (×10^9^/L)	136 (61,186)	204 (150,275)	**<0.001**
Inflammation			
Interleukin 8 (IL 8), pg/mL	154 (85,588)	48 (25, 97)	**<0.001**
Tumor necrosis factor alpha (TNF alpha), pg/mL	56 (40,101)	24 (14, 38)	**<0.001**
Monocyte chemoattractant Protein-1 (MCP 1), pg/mL	1270 (660,2516)	627 (366,1295)	**0.01**
Immunosuppression			
IFN-gamma-inducible protein 10 (IP 10), pg/mL	1287 (619,1935)	558 (333,1132)	**0.003**
Soluble programmed death-ligand 1 (PDL)*, pg/ul	226 (174, 251)	116 (80, 154)	**<0.001**
Bone marrow			
Hemoglobin, g/dL	7.6 (6.9, 9.1)	9.1 (7.7, 10.4)	**0.01**
Red cell distribution width (RDW), %	17 (16, 19)	16 (14, 17)	**<0.001**
Mean corpuscular volume, fL	87 (84, 90)	90 (87, 94)	**0.05**
Stromal cell-derived factor (SDF), pg/mL	3175 (2007,4700)	2357 (1752,2937)	**0.009**
Erythropoietin, (EPO), mIU/mL	55 (23,341)	32 (16, 60)	**0.03**
Metabolism			
Glucagon-like peptide (GLP), pM	281 (97,453)	69 (33,128)	**<0.001**

Data is represented as median (25th percentile, 75th percentile). Pairs that are significant with *P* values at 0.05 level are boldfaced.

* PDL was not used in the analysis to identify clusters.

† Time duration (in min) of the patient where MAP <60 mmHg within the first 24 h from the sepsis onset.
