# Supplementary material for: Early Biomarker Signatures in Surgical Sepsis
Source: J Surg Res. Author manuscript; Available in PMC 2023 Jan 9. (PMC9827429; doi:10.1016/j.jss.2022.04.052)
Supplement: 1 [file NIHMS1852598-supplement-1.docx]

**Supplement Table E1. Biomarkers and other demographic variables used for cluster analysis.**

| **Category** | **Biomarker** | **Units** |
| --- | --- | --- |
| **Demographic** | Age |  |
|  | Charlson comorbidity index |  |
| **Cardiovascular** | Maximum heart rate (beats per minute) within first 24 hours from the sepsis onset |  |
|  | Duration mean arterial pressure (MAP) < 60 mmHg^*^ | minutes |
|  | Brain natriuretic peptide (BNP) | pg/mL |
| **Kidney Function/Acid-base** | Serum Creatinine | mg/dL |
|  | Cystatin C | mg/dL |
|  | Blood urea nitrogen (BUN) | mg/dL |
|  | NephroCheck^Ɨ^ |  |
|  | Fluid overload | % |
|  | Lactate | mmol/L |
|  | Anion Gap | mmol/L |
| **Respiratory** | PaO_2_/FiO_2_ (P/F) ratio |  |
| **Liver function** | Bilirubin | mg/dL |
|  | Aspartate aminotransferase (AST) | U/L |
|  | Alkaline phosphatase | U/L |
| **Endothelial dysfunction/Coagulation** | Angiopoietin 2 (ANG2) | pg/mL |
|  | Fms-related tyrosine kinase (FLT) | pg/mL |
|  | Vascular endothelial growth factor (VEGF) | pg/mL |
|  | International normalized ratio (INR) |  |
|  | Platelet count |  |
| **Inflammation** | Interferon gamma (IFN-gamma) | pg/ml |
|  | Interleukin 6 (IL-6) | pg/ml |
|  | Monocyte chemoattractant protein-1 (MCP 1) | pg/ml |
|  | C-reactive protein (CRP) | mg/L |
|  | Interleukin 8 (IL-8) | pg/ml |
|  | Tumor necrosis factor alpha (TNF-alpha) | pg/ml |
| **Immunosuppression** | Interleukin 10 (IL-10) | pg/ml |
|  | IFN-gamma-inducible protein 10 (IP-10) | pg/ml |
|  | Neutrophils |  |
|  | Neutrophils/Lymphocytes ratio |  |
|  | Lymphocytes |  |
| **Bone marrow** | Hemoglobin | g/dL |
|  | Mean corpuscular volume | fL |
|  | Mean corpuscular hemoglobin concentration (MCHC) | g/dL |
|  | Red cell distribution width (RDW) | % |
|  | Granulocyte-macrophage colony-stimulating factor (GM-CSF) | pg/ml |
|  | Erythropoietin (EPO) | mIU/mL |
|  | Stromal cell-derived factor (SDF) | pg/mL |
| **Catabolism** | Glucose | mg/dL |
|  | Glucagon-like peptide (GLP) | pM |
|  | Insulin-like growth factor-binding protein (IGFBP) | ng/mL |
|  | Insulin-like growth factor (IGF) | ng/mL |
|  | Total protein | g/dL |

^*^ Duration (in minutes) during which mean arterial pressure was < 60 mmHg within the first 24 hours following sepsis onset

^Ɨ^ Urine biomarkers included TIMP-2 and IGFBP7 as part of NephroCheck (1)

1. Vijayan A, Faubel S, Askenazi DJ, Cerda J, Fissell WH, Heung M, Humphreys BD, Koyner JL, Liu KD, Mour G, Nolin TD, Bihorac A, American Society of Nephrology Acute Kidney Injury Advisory G. Clinical Use of the Urine Biomarker [TIMP-2] x [IGFBP7] for Acute Kidney Injury Risk Assessment. *Am J Kidney Dis* 2016; 68: 19-28.
