# Supplementary material for: Early Biomarker Signatures in Surgical Sepsis
Source: J Surg Res. Author manuscript; Available in PMC 2023 Jan 9. (PMC9827429; doi:10.1016/j.jss.2022.04.052)
Supplement: 2 [file NIHMS1852598-supplement-2.docx]

**Supplementary Methods**

# Methods

## Patient Recruitment

Sepsis patients were prospectively recruited between January 2015 and June 2017 from Persistent Inflammation and Immunosuppression in Sepsis (PICS; NCT02276066) prospective longitudinal cohort of surgical patients with sepsis. Study protocols were finalized ^1^ and ethics approval was obtained from the University of Florida Institutional Review Board (IRB201400611) prior to patient enrollment. All study participants or their surrogate decision-maker provided written informed consent. Inclusion criteria for the sepsis cohort were admission to the surgical intensive care unit (ICU), age 18 years or greater, and a clinical diagnosis of sepsis by attending intensivist in surgical ICU with subsequent initiation of the computerized sepsis protocol. ^2^ Patients with pre-existing immunosuppression and those with advanced liver or heart disease were excluded to avoid their potentially confounding effects on the natural history of sepsis and the subsequent development of chronic critical illness ^1^. The final sepsis diagnosis was clinically adjudicated by investigators during weekly adjudication meetings according to consensus criteria. ^3^ Among 243 subjects meeting enrollment criteria with sufficient biomarker data, 157 were included in the development cohort and 86 were included in the validation cohort. Subjects were allocated to development or validation cohorts, based on split in time as illustrated in Figure 1. This study was registered at Clinicaltrials.gov (NCT02276417).

***Generation of Biomarker Signatures***

The clustering algorithms used 58 laboratory and vital sign measurements obtained within 24 hours of sepsis onset, as listed in Supplement Table E1. Measurements that were below detectable limits were assumed to be slightly below the detection limit. Missing values were imputed using the median value of each biomarker for the entire study population. In order to reduce imputation, we only included variables with missingness percentage less than or equal to 4%. Standardized values of 42 biomarkers as well as laboratory values, vital signs, subject age, and Charlson comorbidity index were used to cluster 157 patients into groups with similar clinical and biomarker profiles using an agglomerative hierarchical clustering with complete linkage based on Euclidean distance, i.e., the distance between two points in high-dimensional space. ^4-6^ Agglomerative nesting is a type of hierarchical clustering in which the algorithm starts by treating each object as a singleton cluster, and then pairs of clusters are successively merged until all clusters have been merged into one cluster containing all objects. A dendrogram obtained from hierarchical clustering using the vector of distances denoted by $D_{0}$is shown in Figure 2A. A resampling approach was used to identify clusters by cutting the dendrogram as previously described. ^7,8^ In this resampling method, we generate a reference distribution $D_{ref}$ for $D_{0}$ under the null hypothesis that there would be no significant clusters in the data by randomly mixing the biomarker values of patients for each biomarker and then performing hierarchical clustering on the permuted dataset. We repeat the resampling procedure ten times, and the reference distribution $D_{ref}$ is calculated by taking the average of the distances used for hierarchical clustering in each set. The plot of the observed ($D_{0}$) and expected distances ($D_{ref}$) is displayed in Figure 2C.

***Choice of number of clusters***

Significant clusters were detected by cutting the dendrogram at the 99.6 percentile with α=0.4% of the reference distribution, identifying five clusters which are unlikely to appear in random data. Visual representations as well as clinical characteristics and outcomes of five clusters were observed to determine number of clusters for primary analyses. After investigating the clinical characteristics and outcomes, due to observing only minor differences between two clusters (71 and 64) and in order to avoid even smaller sample sizes spread, we decided to report two main clusters in the primary analysis and keep results of three clusters in supplement. Sensitivity of cluster robustness to different significance levels is illustrated in Figure 2B.

***Leave one feature out analysis***

The dependency of clusters on all variables was tested using ‘leave one feature out’ replication (Supplement Table E2)^7^ in which the cluster analysis was repeated removing one of the 44 biomarkers at a time, and then hierarchical cluster analysis and selection was performed using the same method as in the primary analysis with the significance level of 0.004. Concordance between primary analysis clusters was assessed by Spearman correlations of the cluster labels, summarized in Supplement Table E2, with low correlation indicating change in cluster assignments after exclusion of the feature suggesting importance of feature.

Here we take the cluster concordance correlation which is the correlation between cluster assignments we get with all the features and excluding one feature. If the correlation is high that means we were able to obtain a similar cluster assignment even without the feature that is excluded. Therefore, that particular feature is not important for the clusters we have in hand. If the correlation is low, that means the cluster assignment is different from what we have when we exclude the feature. Therefore, that feature is important to obtain the clusters presented in the paper. In short, a lower correlation is required to claim that all features are important, which is what we observe. The highest correlation we get is just 0.66 when we remove maximum heart rate. This analysis suggested that all biomarkers contributed significantly to primary cluster assignment because excluding any of these biomarkers would result in different cluster assignments.

***Biomarker mosaics***

For each patient, we created a biomarker mosaic using the gene expression dynamics inspector (GEDI) that creates the biomarker mosaics using a self-organizing map (SOM) algorithm.^9,10^ SOM projects the biomarkers onto a 2D mosaic in which the tiles represent individual SOM clusters (typically 1-9 biomarkers) with colors determined by the centroid value of that respective SOM cluster evaluated at each sample. The spatial correspondence of the centroids is preserved due to the mapping of genes to the tiles being invariant across the samples.^9,10^ We compared clinical characteristics and outcomes between the two main clusters using Fisher’s exact test for categorical variables and student’s t-test or Wilcoxon rank sum test for continuous variables as appropriate.

## Definition of Outcomes

All outcomes were compared among clusters and between cohorts. The primary outcomes were in-hospital and one-year mortality. Chronic critical illness (CCI) was defined as an ICU length of stay 14 days or more with evidence of persistent organ dysfunction, determined using components of the SOFA score (cardiovascular SOFA ≥ 1, or score in any other organ system ≥ 2). Non-CCI patients were those who did not meet criteria for CCI or early death (death within 14 days of sepsis onset). ^11^ Other outcomes included hospital-free, ICU-free and mechanical ventilation-free days within 28 days of sepsis onset. Exact dates and times were used to calculate the hospital length of stay, ICU length of stay, and duration of mechanical ventilation. Hospital-free, ICU-free, mechanical ventilation-free, and organ dysfunction free-days within 28 days of sepsis onset were calculated by subtracting the number of days for each of outcome from the lesser of 28 days or the number of days between sepsis onset and death. The Social Security Death Index database was used to confirm death dates and obtain death dates for subjects who were lost to follow-up.

**References**

1. Loftus TJ, Mira JC, Ozrazgat-Baslanti T, et al. Sepsis and Critical Illness Research Center investigators: protocols and standard operating procedures for a prospective cohort study of sepsis in critically ill surgical patients. *BMJ Open.* 2017;7(7):e015136.

2. Croft CA, Moore FA, Efron PA, et al. Computer versus paper system for recognition and management of sepsis in surgical intensive care. *J Trauma Acute Care Surg.* 2014;76(2):311-317; discussion 318-319.

3. American College of Chest Physicians/Society of Critical Care Medicine Consensus Conference: definitions for sepsis and organ failure and guidelines for the use of innovative therapies in sepsis. *Crit Care Med.* 1992;20(6):864-874.

4. Milligan GW, Cooper MC. A study of standardization of variables in cluster analysis. *Journal of Classification.* 1988;Volume 5.

5. Patel S, Sihmar S, Jatain A. A study of hierarchical clustering algorithms. In. 2nd International Conference on Computing for Sustainable Global Development: (INDIACom); 2015:pp. 537-541.

6. Sasirekha K, Baby P. Agglomerative Hierarchical Clustering Algorithm-A Review. *International Journal of Scientific and Research Publications.* 2013;Volume 3(Issue 3).

7. Sebastiani P, Perls TT. Detection of Significant Groups in Hierarchical Clustering by Resampling. *Front Genet.* 2016;7:144.

8. Kumar A, Roberts D, Wood KE, et al. Duration of hypotension before initiation of effective antimicrobial therapy is the critical determinant of survival in human septic shock. *Crit Care Med.* 2006;34(6):1589-1596.

9. Wong HR, Cvijanovich NZ, Allen GL, et al. Validation of a gene expression-based subclassification strategy for pediatric septic shock. *Crit Care Med.* 2011;39(11):2511-2517.

10. Eichler GS, Huang S, Ingber DE. Gene Expression Dynamics Inspector (GEDI): for integrative analysis of expression profiles. *Bioinformatics.* 2003;19(17):2321-2322.

11. Stortz JA, Mira JC, Raymond SL, et al. Benchmarking clinical outcomes and the immunocatabolic phenotype of chronic critical illness after sepsis in surgical intensive care unit patients. *J Trauma Acute Care Surg.* 2018;84(2):342-349.
