# Supplementary material for: Early Biomarker Signatures in Surgical Sepsis
Source: J Surg Res. Author manuscript; Available in PMC 2023 Jan 9. (PMC9827429; doi:10.1016/j.jss.2022.04.052)
Supplement: 3 [file NIHMS1852598-supplement-3.docx]

**Supplement Table E2. Results of the leave-one-biomarker-out replication.**

| **Biomarker dropped** | **Correlation** | **Biomarker dropped** | **Correlation** |
| --- | --- | --- | --- |
| GM CSF | 0.17 | INR | 0.17 |
| IFN gamma | 0.51 | Neutrophils/Lymphocytes ratio | -0.09 |
| IL 6 | 0.07 | Total protein | 0.18 |
| IL 8 | -0.32 | Bilirubin | 0.19 |
| IL 10 | -0.06 | Alkaline phosphatase | 0.19 |
| IP 10 | 0.3 | AST | 0.23 |
| MCP 1 | 0.04 | Glucose | 0.18 |
| TNF alpha | 0.14 | BUN | 0.41 |
| VEGF | 0.41 | Anion Gap | 0.33 |
| ANG2 | -0.12 | Hemoglobin | -0.19 |
| EPO | 0.44 | Mean corpuscular volume | 0.24 |
| FLT | -0.14 | MCHC | 0.33 |
| GLP | 0.27 | CRP | 0.51 |
| SDF | 0.07 | Nephrocheck | 0.54 |
| IGFBP | 0.12 | Age | 0.58 |
| IGF | 0.41 | Charlson comorbidity index | 0.43 |
| Neutrophils | 0.19 | Maximum heart rate | **0.66** |
| Serum creatinine | -0.13 | Fluid overload | 0.16 |
| Platelet | **-0.03** | P/F ratio | 0.25 |
| BNP | 0.4 | RDW | 0.09 |
| Cystatin C | -0.17 | Duration MAP < 60 mmHg | 0.48 |
| Lactate | 0.18 | Lymphocytes | 0.48 |
