# Supplementary material for: Early Biomarker Signatures in Surgical Sepsis
Source: J Surg Res. Author manuscript; Available in PMC 2023 Jan 9. (PMC9827429; doi:10.1016/j.jss.2022.04.052)
Supplement: 4 [file NIHMS1852598-supplement-4.doc]

**Supplement Table E3. Clinical characteristics of patients in the validation cohort.**

|  | **Cluster I**  Early disrupted homeostasis  **(N=29)** | **Cluster II**  Early preserved homeostasis  **(N=57)** | **P value** |
| --- | --- | --- | --- |
| **Demographics** |  |  |  |
| Age, median (25th, 75th) | 64 (58, 73) | 59 (47, 72) | 0.16 |
| Male gender, n (%) | 19 (66) | 28 (49) | 0.17 |
| Race, n (%) |  |  | 0.22 |
| White | 25 (86) | 54 (95) |  |
| African American | 4 (14) | 53 (5) |  |
| American Indian | 0 (0) | 0(0) |  |
| Transferred from another hospital, n (%) | 15 (52) | 25 (44) | 0.50 |
| Weight (kg) , median (25th, 75th) | 96 (74, 123) | 85 (74, 105) | 0.34 |
| Body Mass Index, median (25th, 75th) | 33 (25, 41) | 30 (25, 36) | 0.38 |
| **Comorbidities, n (%)** |  |  |  |
| Chronic Kidney Disease (CKD), n (%)^*^ | 5 (19) | 9 (16) | 0.76 |
| Congestive Heart Failure, n (%) | 3 (10) | 1 (2) | 0.12 |
| Chronic obstructive pulmonary disease, n (%)^Ɨ^ | 2 (7) | 4 (7) | 1.00 |
| Diabetes mellitus, n (%) | 12 (41) | 9 (16) | **0.02** |
| Hypertension, n (%)^Ɨ^ | 20 (69) | 27 (48) | 0.12 |
| Smoking History, n (%)^Ɨ^ |  |  | 0.11 |
| Current | 12 (41) | 14 (25) |  |
| Former | 3 (10) | 6 (11) |  |
| Never | 14 (48) | 36 (64) |  |
| **Characteristics of Sepsis episode** |  |  |  |
| Sepsis Severity, n (%) |  |  | **<0.001** |
| Sepsis | 1 (4) | 20 (35) |  |
| Severe Sepsis | 14 (48) | 30 (53) |  |
| Septic Shock | 14 (48) | 7 (12) |  |
| Anatomic site of infection, n (%) |  |  | 0.52 |
| Abdominal | 15 (52) | 19 (33) |  |
| Pulmonary | 5 (17) | 9 (16) |  |
| Skin/Soft Tissue | 3 (10) | 12 (21) |  |
| Genitourinary | 4 (14) | 11 (19) |  |
| Vascular | 2 (7) | 6 (11) |  |
| APACHE II within first 24 hours of sepsis onset, median (25th, 75th) | 25 (21, 29) | 17 (11, 20) | **<0.001** |
| Total Acute Physiology Score | 20 (14, 26) | 14 (8, 17) | **<0.001** |
| Age Points | 3 (3, 5) | 3 (2, 5) | 0.27 |
| Chronic health points | 0 (0, 0) | 0 (0, 0) | **0.01** |
| SOFA score on sepsis onset day, median (25th, 75th) | 9 (7, 12) | 5 (3, 7) | **<0.001** |
| Respiratory system | 1 (0, 3) | 1 (0, 2) | 0.60 |
| Central nervous system | 2 (1, 3) | 1 (0, 2) | **0.04** |
| Cardiovascular system | 3 (1, 4) | 1 (1, 1) | **<0.001** |
| Liver | 0 (0, 1) | 0 (0, 0) | 0.16 |
| Coagulation | 0 (0, 1) | 0 (0, 1) | **0.02** |
| Renal | 3 (2, 4) | 0 (0, 1) | **<0.001** |

Abbreviations. APACHE II, Acute Physiology and Chronic Health Evaluation II score; SOFA, Sequential Organ Failure Assessment.

^*^ Percentages calculated after removing ESRD patients from the cohort.

^Ɨ^ Due to missing values percentages were calculated based on available.

Pairs that are significant with p values at 0.05 level are boldfaced.
