# Supplementary material for: Early Biomarker Signatures in Surgical Sepsis
Source: J Surg Res. Author manuscript; Available in PMC 2023 Jan 9. (PMC9827429; doi:10.1016/j.jss.2022.04.052)
Supplement: 5 [file NIHMS1852598-supplement-5.docx]

**Supplement Table E4. Outcome characteristics of patients in the validation cohort.**

| **Clinical Outcome** | **Cluster I**  Early  disrupted homeostasis  **(N=28)^*^** | **Cluster II**  Early preserved homeostasis  **(N=56)^*^** | **P value** |
| --- | --- | --- | --- |
| Hospital mortality, n (%) | 9 (32) | 6 (11) | **0.03** |
| One year mortality, n (%)^Ɨ ǂ^ | 16/27 (59) | 7/53 (13) | **<0.001** |
| One year mortality among survivors, n (%)^Ɨ ǂ^ | 7/18 (39) | 1/47 (2) | **<0.001** |
| Chronic critical illness (CCI), n (%) |  |  | **<0.001** |
| Early Death | 6 (21) | 1 (2) |  |
| CCI | 15 (54) | 14 (25) |  |
| Non-CCI | 7 (25) | 41 (73) |  |
| Kidney disease, n (%) | 28 (100) | 39 (70) | **<0.001** |
| ESKD | 3 (11) | 0 (0) |  |
| CKD, no AKI | 0 (0) | 1 (2) |  |
| CKD and AKI | 4 (14) | 8 (14) |  |
| AKI, no CKD | 21 (75) | 30 (54) |  |
| No renal disease | 0 (0) | 17 (30) |  |
| Acute Kidney Injury severity, n (%) |  |  | **<0.001** |
| Stage 1 | 1 (4) | 19 (34) |  |
| Stage 2 | 8 (29) | 11 (20) |  |
| Stage 3 | 16 (57) | 8 (14) |  |
| Renal replacement therapy (RRT), n (%) | 12 (43) | 5 (9) | **<0.001** |
| Duration of RRT(days)^§^, median (25th, 75th) | 12 (3, 33) | 16 (10, 18) | 0.63 |
| RRT-free days to day 28, median (25th, 75th) | 21 (0, 28) | 28 (28, 28) | **<0.001** |
| Renal recovery at discharge, n (%)^\|\|^ | 13 (52) | 26 (68) | 0.29 |
| Hospital days, median (25th, 75th) | 23 (7, 38) | 15 (9, 23) | 0.42 |
| Hospital-free days to day 28, median (25th, 75th) | 0 (0, 3) | 10 (0, 18) | **0.005** |
| Days in Intensive Care Unit, median (25th, 75th) | 16 (3, 25) | 6 (3, 12) | **0.03** |
| ICU-free days to day 28, median (25th, 75th) | 2 (0, 16) | 22 (16, 25) | **<0.001** |
| Need for mechanical ventilation, n (%) | 22 (79) | 32 (57) | 0.06 |
| Days on mechanical ventilator^¶^, median (25th, 75th) | 8 (3, 16) | 5 (3, 12) | **0.04** |
| MV-free days to day 28, median (25th, 75th) | 18 (0, 25) | 27 (23, 28) | **0.001** |
| SOFA organ dysfunction-free days to day 28, median (25th, 75th) | 1 (0, 18) | 21 (11, 25) | **<0.001** |
| Discharged home, n (%) | 9 (32) | 35 (63) | **0.011** |
| Readmission or death within 30 days of initial discharge, n (%) | 8 (29) | 12 (21) | 0.59 |

Abbreviations: ESKD, end-stage kidney disease; CKD, chronic kidney disease; CCI, chronic critical illness; LOS, length of stay; ICU, Intensive Care Unit; MV, mechanical ventilation; RRT, renal replacement therapy.

Pairs that are significant with p values at 0.05 level are boldfaced.

^*^ Hospital outcome data were not available for some patients due to withdrawal of patients from study before hospital discharge.

^Ɨ^ Due to missing values percentages were calculated based on available.

^ǂ^ Twelve-month data were not available for some patients due to withdrawal of patients from study before 12-month follow-up.

^§^ among patients who required RRT

^||^ among patients who had AKI

^¶^ among patients who required mechanical ventilation
