# Supplementary material for: Early Biomarker Signatures in Surgical Sepsis
Source: J Surg Res. Author manuscript; Available in PMC 2023 Jan 9. (PMC9827429; doi:10.1016/j.jss.2022.04.052)
Supplement: 6 [file NIHMS1852598-supplement-6.doc]

**Supplement Table E5. Characteristics of biomarkers available within 24 hours of sepsis in two clusters in the validation cohort.**

| **Biomarkers within 24 hours of sepsis onset** | **Cluster I**  Early disrupted homeostasis  **(N=29)** | **Cluster II**  Early preserved homeostasis  **(N=57)** | **P value** |
| --- | --- | --- | --- |
| **Cardiovascular** |  |  |  |
| Brain natriuretic peptide (BNP), pg/mL | 2465 (1368, 7628) | 596 (300, 1889) | **<0.001** |
| Duration mean arterial pressure (MAP) < 60, mmHg (minutes)^*^ | 120 (15, 275) | 60 (0, 122) | **0.01** |
| Maximum heart rate (beats per minute) within first 24 hours from the sepsis onset | 115 (106, 125) | 125 (106, 132) | 0.20 |
| **Kidney** |  |  |  |
| Serum Creatinine, mg/dL | 2.34 (1.71, 3.6) | 1.01 (0.71, 1.35) | **<0.001** |
| Cystatin C, mg/dL | 2 (1.3, 2.6) | 0.9 (0.7, 1.1) | **<0.001** |
| Blood urea nitrogen (BUN), mg/dL | 46 (30, 61) | 19 (14, 27) | **<0.001** |
| Anion Gap, mmol/L | 21 (19, 25) | 16 (14, 19) | **<0.001** |
| Fluid overload, % | 12 (6, 27) | 6 (3, 10) | **0.003** |
| Lactate, mmol/L | 3.4 (2.2, 6.5) | 2 (1.4, 3.1) | **0.002** |
| Nephrocheck | 0.22 (0.11, 0.32) | 0.31 (0.16, 1.08) | 0.25 |
| **Respiratory** |  |  |  |
| Ratio of partial pressure arterial oxygen and fraction of inspired oxygen (PaO2/FiO2), mmHg | 272 (131, 457) | 269 (177, 437) | 1.00 |
| **Liver** |  |  |  |
| Bilirubin, mg/dL | 0.7 (0.5, 1.5) | 0.8 (0.4, 1.05) | 0.36 |
| Aspartate Aminotransferase (AST) Test (SGOT), U/L | 42 (29, 75) | 31 (20, 46) | **0.01** |
| **Endothelial function and coagulation** |  |  |  |
| Angiopoietin-2 (Ang2), ng/mL | 19 (11, 26) | 9 (6, 11) | **<0.001** |
| Fms Related Tyrosine (Flt), pg/mL | 439 (272, 1808) | 186 (141, 301) | **<0.001** |
| International Normalized Ratio (INR) | 1.6 (1.4, 2.2) | 1.4 (1.2, 1.5) | **<0.001** |
| Platelet count (x10^9^/L) | 144 (94, 226) | 219 (138, 305) | **0.004** |
| **Inflammation** |  |  |  |
| Interleukin 8 (IL 8), pg/ml | 172 (121, 245) | 43 (23, 104) | **<0.001** |
| Tumor necrosis factor alpha (TNF alpha), pg/ml | 118 (58, 193) | 70 (57, 93) | **0.04** |
| Monocyte Chemoattractant Protein-1 (MCP 1), pg/ml | 1004 (734, 2536) | 679 (416, 1301) | **0.002** |
| **Immunosuppression** |  |  |  |
| IFN-gamma-inducible protein 10 (IP 10), pg/ml | 933 (686, 2181) | 893 (393, 1533) | 0.12 |
| Soluble programmed death-ligand 1 (PDL)^Ɨ^, pg/ul | 212 (121, 254) | 133 (102, 180) | **0.02** |
| **Bone marrow** |  |  |  |
| Hemoglobin, g/dL | 8.7 (7.2, 9.8) | 9.2 (7.8, 10.5) | 0.23 |
| Red cell distribution width (RDW), % | 16 (15, 18) | 15 (14, 16) | **0.002** |
| Mean Corpuscular Volume, fL | 88 (85, 94) | 87 (84, 93) | 0.50 |
| Stromal cell-derived factor (SDF), pg/mL | 3699 (2888, 5142) | 3063 (2252, 4235) | **0.03** |
| Erythropoietin, (EPO), mIU/mL | 38 (24, 113) | 32 (18, 54) | 0.12 |
| **Metabolism** |  |  |  |
| Glucagon-like peptide (GLP), pM | NA | NA | NA |

Data is represented as median (25th percentile, 75th percentile).

Pairs that are significant with p values at 0.05 level are boldfaced.

^*^ Time duration (in minutes) of the patient where MAP < 60 mmHg within the first 24 hours from the sepsis onset.

^Ɨ^ PDL was not used in the analysis to identify clusters.
