# Supplementary material for: Early Biomarker Signatures in Surgical Sepsis
Source: J Surg Res. Author manuscript; Available in PMC 2023 Jan 9. (PMC9827429; doi:10.1016/j.jss.2022.04.052)
Supplement: 7 [file NIHMS1852598-supplement-7.doc]

**Supplement Table E6. Clinical characteristics of patients in development and validation cohorts.**

|  | **Development Cohort (N=157)** | **Validation Cohort**  **(N=86)** | **P value** |
| --- | --- | --- | --- |
| **Demographics** |  |  |  |
| Age, median (25th, 75th) | 62 (51, 70) | 62 (50, 72) | 0.84 |
| Male gender, n (%) | 85 (54) | 47 (55) | 1 |
| Race, n (%) |  |  | 0.95 |
| White | 139 (89) | 79 (92) |  |
| African American | 13 (8) | 7 (8) |  |
| American Indian | 1 (1) |  |  |
| Transferred from another hospital, n (%) | 72 (46) | 40 (47) | 1 |
| Weight (kg), median (25th, 75th) | 86 (70, 104) | 87 (74, 112) | 0.30 |
| Body Mass Index, median (25th, 75th) | 29 (25, 36) | 30 (25, 38) | 0.28 |
| **Comorbidities, n (%)** |  |  |  |
| Chronic Kidney Disease^*^ | 22 (14) | 14 (17) | 0.70 |
| Congestive Heart Failure | 27 (17) | 4 (5) | **<0.01** |
| Chronic obstructive pulmonary disease^Ɨ^ | 19 (12) | 6 (7) | 0.27 |
| Diabetes mellitus^Ɨ^ | 53 (34) | 21 (24) | 0.14 |
| Hypertension^Ɨ^ | 94 (61) | 47 (55) | 0.41 |
| Smoking History, n (%) |  |  | 0.32 |
| Current | 61 (39) | 26 (30) |  |
| Former | 16 (10) | 9 (11) |  |
| Never | 76 (48) | 50 (58) |  |
| **Characteristics of Sepsis episode** |  |  |  |
| Sepsis Severity, n (%) |  |  | 0.13 |
| Sepsis | 57 (36) | 21 (24) |  |
| Severe Sepsis | 62 (39) | 44 (51) |  |
| Septic Shock | 38 (24) | 21 (24) |  |
| **Acuity Scores in the first 24 hours of sepsis onset,** median (25th, 75th) |  |  |  |
| APACHE II | 17 (12, 23) | 19 (14, 24) | 0.09 |
| Total Acute Physiology Score | 13 (8, 19) | 15 (9, 20) | 0.12 |
| Age Points | 3 (2, 5) | 3 (2, 5) | 0.58 |
| Chronic health points | 0 (0, 0) | 0 (0, 0) | 0.40 |
| SOFA | 6 (3, 9) | 6 (4, 9) | 0.21 |
| Respiratory system | 1 (0, 3) | 1 (0, 3) | 0.41 |
| Central nervous system | 2 (0, 3) | 1 (0, 3) | 0.92 |
| Cardiovascular system | 1 (1, 3) | 1 (1, 3) | 0.10 |
| Liver | 0 (0, 1) | 0 (0, 1) | 0.93 |
| Coagulation | 0 (0, 0) | 0 (0, 1) | 0.21 |
| Renal | 0 (0, 1) | 1 (0, 3) | **0.02** |

Abbreviations. APACHE II, Acute Physiology and Chronic Health Evaluation II score; SOFA, Sequential Organ Failure Assessment.

^*^ Percentages calculated after removing ESRD patients from the cohort.

^Ɨ^ Due to missing values percentages were calculated based on available.

Pairs that are significant with p values at 0.05 level are boldfaced.
