# Supplementary material for: Early Biomarker Signatures in Surgical Sepsis
Source: J Surg Res. Author manuscript; Available in PMC 2023 Jan 9. (PMC9827429; doi:10.1016/j.jss.2022.04.052)
Supplement: 8 [file NIHMS1852598-supplement-8.docx]

**Supplement Table E7. Outcome characteristics of patients in development and validation cohorts.**

| **Clinical Outcomes** | **Development Cohort (N=149)^*^** | **Validation Cohort**  **(N=84)^*^** | **P value** |
| --- | --- | --- | --- |
| Hospital mortality, n (%) | 12 (8) | 15 (18) | **0.03** |
| One year mortality, n (%)^Ɨ ǂ^ | 32/140 (23) | 23/80 (29) | 0.34 |
| One year mortality among survivors, n (%)^ǂ^ | 20/128 (16) | 8/65 (12) | 0.67 |
| Chronic critical illness (CCI), n (%) |  |  | 0.13 |
| Early Death | 4 (3) | 7 (8) |  |
| CCI | 50 (34) | 29 (35) |  |
| Non-CCI | 95 (64) | 48 (57) |  |
| Kidney disease, n (%) |  |  | **<0.01** |
| ESKD | 3 (2) | 3 (4) |  |
| CKD, no AKI | 5 (3) | 1 (1) |  |
| AKI and CKD | 17 (11) | 12 (14) |  |
| AKI, no CKD | 59 (40) | 51 (61) |  |
| No renal disease | 65 (44) | 17 (20) |  |
| Acute Kidney Injury severity, n (%) |  |  | **<0.01** |
| Stage 1 | 35 (24) | 20 (24) |  |
| Stage 2 | 23 (15) | 19 (23) |  |
| Stage 3 | 18 (12) | 24 (29) |  |
| Renal replacement therapy (RRT), n (%) | 13 (9) | 17 (20) | **0.01** |
| Duration of RRT (days)^§^, median (25th, 75th) | 13 (8, 29) | 16 (4, 31) |  |
| RRT-free days to day 28, median (25th, 75th) | 28 (28, 28) | 28 (20.5, 28) | **<0.01** |
| Renal recovery at discharge, n (%)^\|\|^ | 46 (61) | 39 (62) | 1 |
| Hospital days, median (25th, 75th) | 17 (8, 28) | 16 (8, 31) | 0.92 |
| Hospital-free days to day 28, median (25th, 75th) | 9 (0, 18) | 3.5 (0, 16) | 0.13 |
| Days in Intensive Care Unit, median (25th, 75th) | 7 (4, 17) | 7 (3, 19.5) | 0.65 |
| ICU-free days to day 28, median (25th, 75th) | 20 (11, 24) | 19.5 (2, 25) | 0.40 |
| Need for mechanical ventilation, n (%) | 105 (70) | 54 (64) | 0.38 |
| Days on mechanical ventilator^¶^, median (25th, 75th) | 5 (3, 10) | 6 (3, 15) | 0.43 |
| MV-free days to day 28, median (25th, 75th) | 25 (20, 28) | 25 (14, 28) | 0.86 |
| SOFA organ dysfunction-free days to day 28, median (25th, 75th) | 18 (8, 23) | 19 (1, 23) | 0.48 |
| Discharged home, n (%) | 73 (49) | 44 (52) | 0.68 |
| Readmission or death within 30 days of initial discharge, n (%) | 36 (24) | 20 (24) | 1.00 |

Abbreviations: ESKD, end-stage kidney disease; CKD, chronic kidney disease; CCI, chronic critical illness; ICU, intensive care unit; MV, mechanical ventilation; RRT, renal replacement therapy; SOFA, Sequential Organ Failure Assessment.

Pairs that are significant with p values at 0.05 level are boldfaced.

^*^ Hospital outcome data were not available for some patients due to withdrawal of patients from study before hospital discharge.

^Ɨ^ Due to missing values percentages were calculated based on available.

^ǂ^ Twelve-month data were not available for some patients due to withdrawal of patients from study before 12-month follow-up.

^§^ among patients who required RRT.

^||^ among patients who had AKI.

^¶^ among patients who required mechanical ventilation.
