# Supplementary material for: Early Biomarker Signatures in Surgical Sepsis
Source: J Surg Res. Author manuscript; Available in PMC 2023 Jan 9. (PMC9827429; doi:10.1016/j.jss.2022.04.052)
Supplement: 9 [file NIHMS1852598-supplement-9.doc]

**Supplement Table E8. Characteristics of biomarkers available within 24 hours of sepsis onset in development and validation cohorts.**

| **Biomarkers within 24 hours of sepsis onset** | **Development Cohort (N=157)** | **Validation Cohort (N=86)** | **P value** |
| --- | --- | --- | --- |
| **Cardiovascular** |  |  |  |
| Brain natriuretic peptide (BNP), pg/mL | 1192 (383, 3326) | 1238 (470, 3161) | 0.76 |
| Duration mean arterial pressure (MAP) < 60, mmHg (minutes)^*^ | 30 (0, 120) | 60 (0, 180) | 0.09 |
| Maximum heart rate (beats per minute) within first 24 hours from the sepsis onset | 120 (108, 132) | 121 (106, 132) | 0.83 |
| **Kidney** |  |  |  |
| Serum Creatinine, mg/dL | 1.0 (0.8, 1.6) | 1.2 (0.8, 2.2) | **0.02** |
| Cystatin C, mg/dL | 0.9 (0.6, 1.2) | 1.1 (0.8, 1.6) | **0.005** |
| Blood urea nitrogen (BUN), mg/dL | 20 (13, 31) | 26 (15, 38) | 0.07 |
| Anion Gap, mmol/L | 17 (14, 20) | 18 (14, 21) | 0.28 |
| Fluid overload, % | 7 (3, 11) | 7 (4, 12) | 0.28 |
| Lactate, mmol/L | 1.9 (1.3, 3.2) | 2.4 (1.5, 3.8) | **0.04** |
| Nephrocheck | 0.38 (0.18, 1.17) | 0.29 (0.16, 0.92) | 0.36 |
| **Respiratory** |  |  |  |
| Ratio of partial pressure arterial oxygen and fraction of inspired oxygen (PaO2/FiO2), mmHg | 327 (188, 454) | 272 (164, 440) | 0.18 |
| **Liver** |  |  |  |
| Bilirubin, mg/dL | 0.7 (0.4, 1.3) | 0.8 (0.5, 1.2) | 0.87 |
| Aspartate Aminotransferase (AST) Test (SGOT), U/L | 26 (19, 54) | 34 (22, 54) | 0.16 |
| **Endothelial function and coagulation** |  |  |  |
| Angiopoietin-2 (Ang2), ng/mL | 8 (5, 12) | 10 (7, 16) | **0.01** |
| Fms Related Tyrosine (Flt), pg/mL | 189 (133, 306) | 259 (153, 443) | **0.01** |
| International Normalized Ratio (INR) | 1.4 (1.3, 1.7) | 1.4 (1.2, 1.6) | 0.67 |
| Platelet count (x109/L) | 199 (140, 263) | 160.5 (123, 268) | 0.27 |
| **Inflammation** |  |  |  |
| Interleukin 8 (IL 8), pg/ml | 58 (26, 118) | 85 (31, 172) | 0.13 |
| Tumor necrosis factor alpha (TNF alpha), pg/ml | 26 (17, 45) | 76 (57, 139) | **<0.0001** |
| Monocyte Chemoattractant Protein-1 (MCP 1), pg/ml | 663 (391, 1360) | 745 (478, 1693) | 0.08 |
| **Immunosuppression** |  |  |  |
| IFN-gamma-inducible protein 10 (IP 10), pg/ml | 619 (355, 1207) | 897 (421, 1642) | **0.02** |
| Soluble programmed death-ligand 1 (PDL)^Ɨ^, pg/ul | 122 (84, 164) | 147 (108, 214) | **0.0008** |
| **Bone marrow** |  |  |  |
| Hemoglobin, g/dL | 9.0 (7.6, 10.4) | 8.8 (7.6, 10.4) | 0.93 |
| Red cell distribution width (RDW), % | 16 (15, 17) | 15 (14, 17) | 0.17 |
| Mean Corpuscular Volume, fL | 90 (87, 94) | 88 (85, 94) | 0.07 |
| Stromal cell-derived factor (SDF), pg/mL | 2403 (1770, 3068) | 3304 (2609, 4598) | **<0.0001** |
| Erythropoietin, (EPO), mIU/mL | 32 (17, 62) | 35 (19, 59) | 0.84 |
| **Metabolism** |  |  |  |
| Glucagon-like peptide (GLP), pM | 81 (38, 149) | NA |  |

Data is represented as median (25th percentile, 75th percentile).

Pairs that are significant with p values at 0.05 level are boldfaced.

^*^ Time duration (in minutes) of the patient where MAP < 60 mmHg within the first 24 hours from the sepsis onset.

^Ɨ^ PDL was not used in the analysis to identify clusters.
