# Supplementary figures and images for: Early Biomarker Signatures in Surgical Sepsis
Source: J Surg Res. Author manuscript; Available in PMC 2023 Jan 9. (PMC9827429; doi:10.1016/j.jss.2022.04.052)

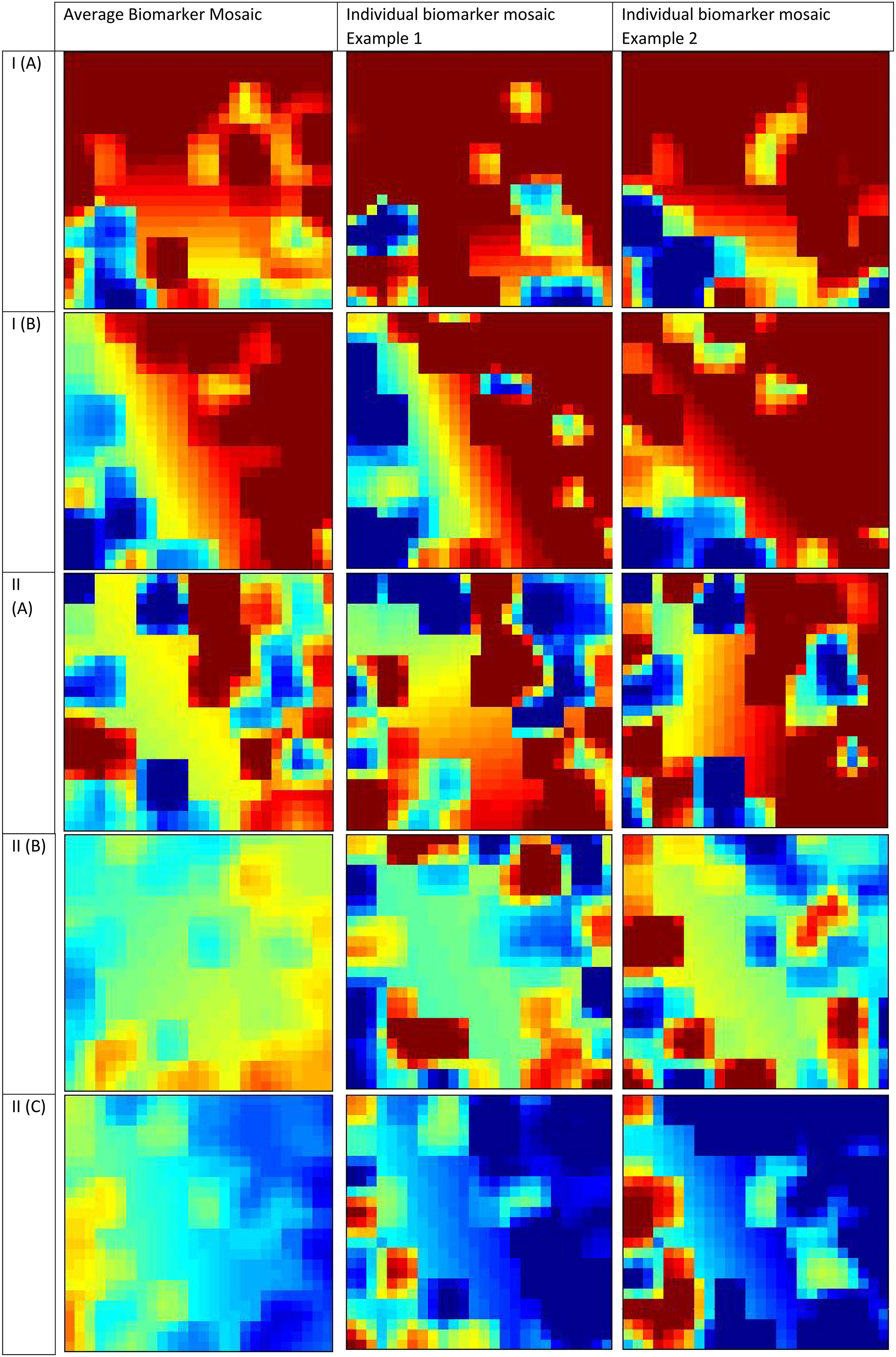

Supplement: figsE4 [file NIHMS1852598-supplement-figsE4.jpg]

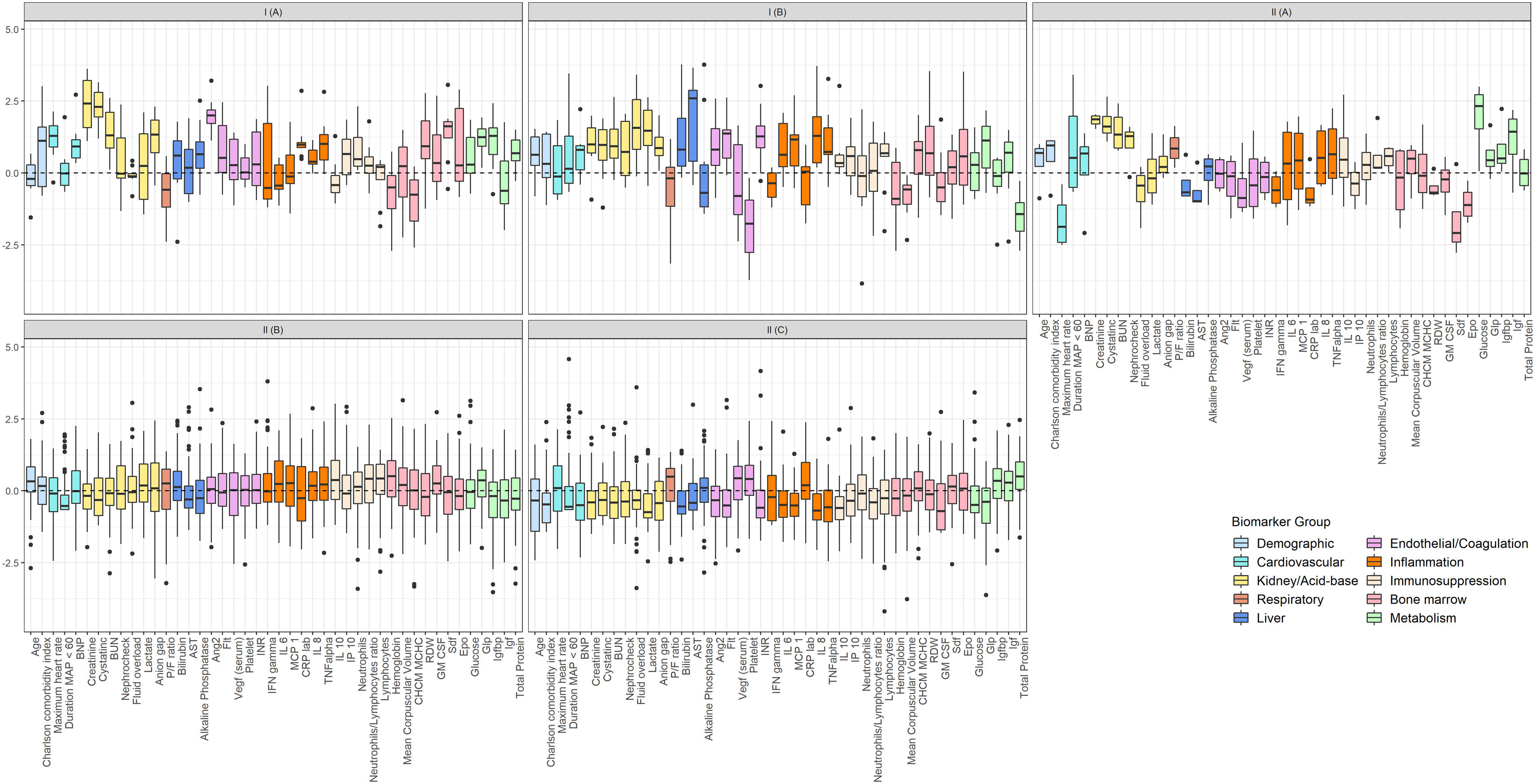

Supplement: figsE3 [file NIHMS1852598-supplement-figsE3.jpg]

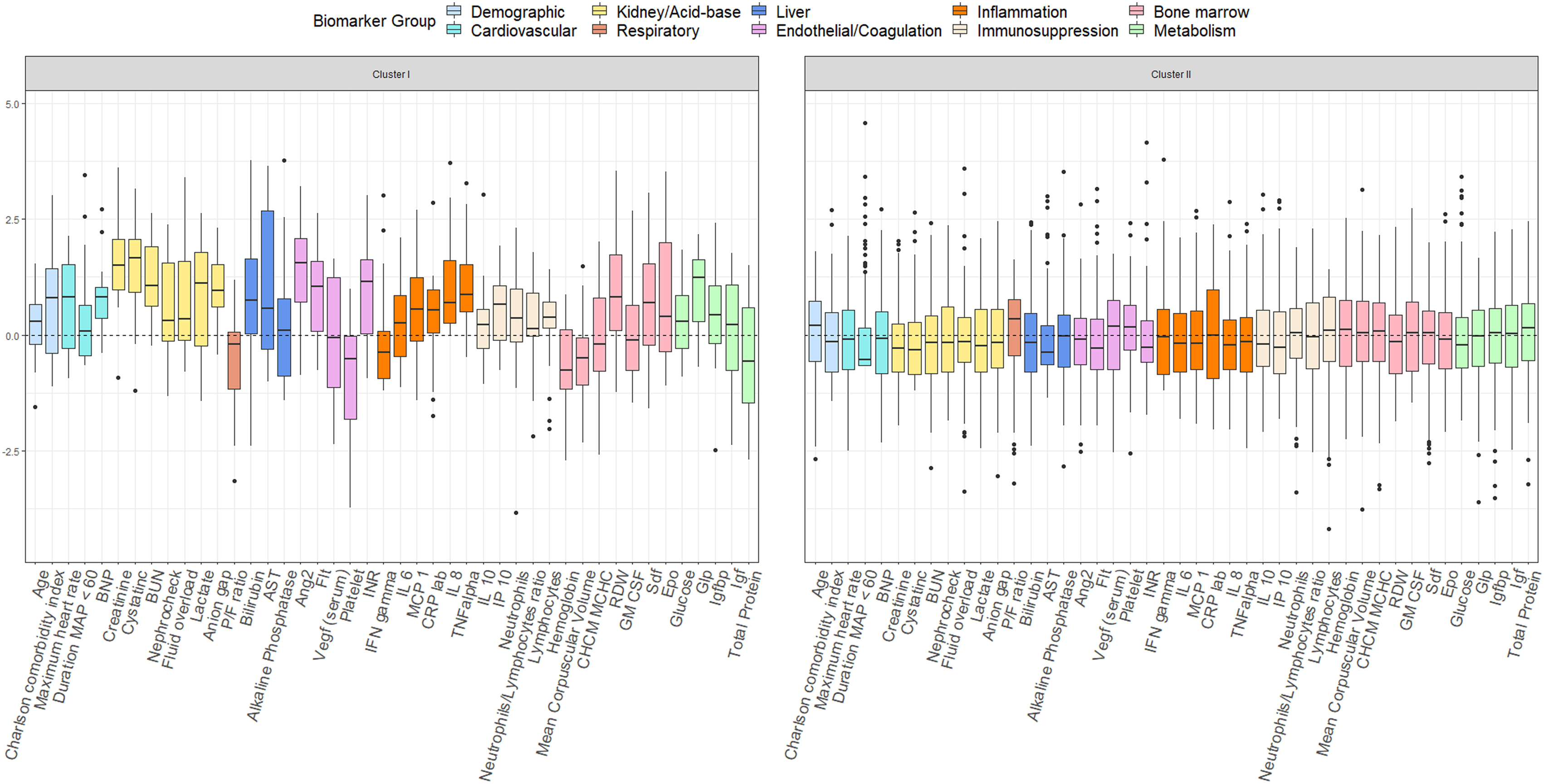

Supplement: figsE2 [file NIHMS1852598-supplement-figsE2.jpg]

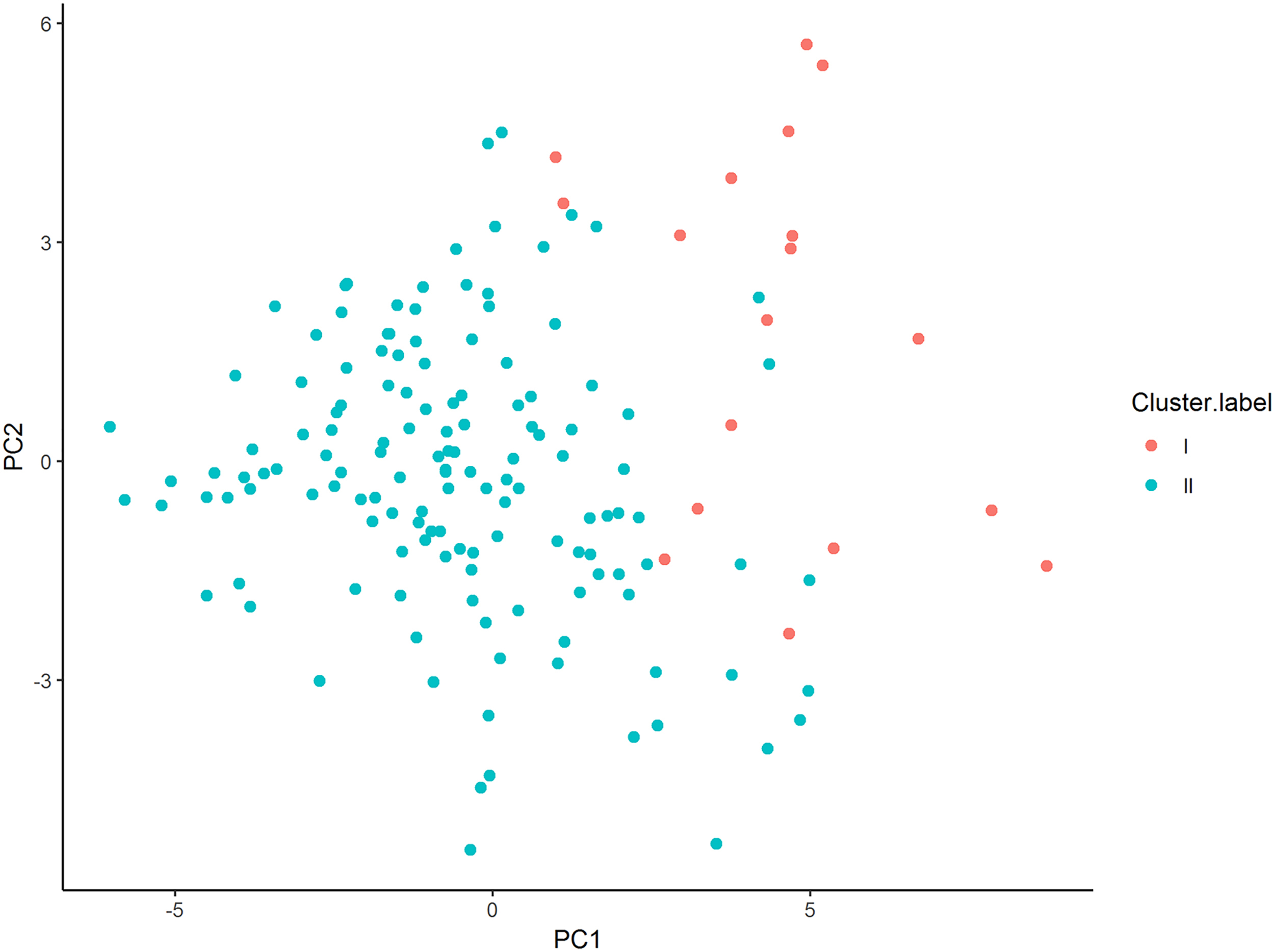

Supplement: figsE1 [file NIHMS1852598-supplement-figsE1.jpg]
